# Single-Cell *cis*-Mendelian Randomization Reveals Cell-Specific Genetic Mechanisms Underlying Atopic Dermatitis

**DOI:** 10.3390/ijms27052226

**Published:** 2026-02-27

**Authors:** Charalabos Antonatos, Yiannis Vasilopoulos

**Affiliations:** Laboratory of Genetics, Section of Genetics, Cell Biology and Development, Department of Biology, University of Patras, 26504 Patras, Greece; charisantonatos@gmail.com

**Keywords:** atopic dermatitis, eczema, single cell, Mendelian Randomization, transcriptome-wide association study, eQTL

## Abstract

Atopic dermatitis (AD) is a chronic inflammatory skin disease with a complex and highly polygenic genetic architecture, in which immune-mediated mechanisms play a central role. Here, we integrated single-cell *cis*-expression quantitative trait loci from 14 immune cell types with AD GWAS summary statistics using a two-sample Mendelian Randomization (MR) framework to resolve cell-specific genetically mediated transcriptional effects. We identified 303 significant cell-specific gene–trait associations with limited overlaps across cell types. A multi-step prioritization strategy refined these findings to 35 genes across all 14 cell types. A comparison with whole blood *cis*-eQTLs revealed a limited concordance, suggesting an attenuation of cell-specific regulatory effects in bulk transcriptomic approaches. Intersecting single-cell and bulk evidence identified 22 high-confidence genes with a relatively independent mechanism of action. Integrative annotation implicated several immune-relevant and druggable genes, including *IL2RA*, with distinct cell-specific effects. Our findings demonstrate diverse mechanisms of risk genes for AD at the single-cell level that act across immune cell states and pathways, with implications for therapeutic interventions.

## 1. Introduction

Atopic dermatitis (AD), or eczema, is a common inflammatory cutaneous disease characterized by clinical heterogeneity and a complex genetic architecture [1]. Twin and family-based studies estimate heritability to be as high as 80% [1], with genome-wide association studies (GWAS) additionally identifying numerous genomic risk loci contributing to disease susceptibility [2]. Although early genetic discoveries supported AD as a predominantly skin barrier-driven model of disease given the loss-of-function variants in the *FLG* gene, findings from large-scale genomic studies have demonstrated an important role for immune dysregulation in the onset and maintenance of allergic skin reactions, as well as associated allergic [3] and non-allergic [4,5] comorbidities. Despite these advances, translating genomic signals in mechanistic insights and potential novel therapeutic avenues remains challenging given (i) the enrichment of associated variants in non-coding regions [6] and (ii) the context-dependent nature of regulatory effects [7].

To bridge this gap, various omics-based frameworks have been suggested and successfully applied in complex diseases to understand their heritable background. One of the most common frameworks in post-GWAS analyses is the employment of expression quantitative trait loci (eQTLs) [8], linking genetic variation to gene regulation and hence prioritizing candidate risk genes. Nevertheless, most existing studies rely on bulk transcriptomic profiles, which aggregate regulatory effects across heterogeneous cell populations with distinct and sometimes opposing roles in disease pathogenesis. In AD, we previously leveraged bulk *cis*-eQTL resources across three relevant tissues, identifying more than 100 candidate genes, and, by integrating bulk RNA sequencing studies, we prioritized 16 hub genes involved in skin-related processes in AD lesional skin [9]. While informative, such bulk-based approaches are inherently limited in their ability to resolve cell-specific regulatory mechanisms. This is of particular interest in immune-driven diseases, as is the case with AD, due to the context-dependent, distinct regulatory transcriptional programs that govern each cell type.

Higher resolution analyses could provide a more detailed view of the genetic architecture underlying transcriptional activity in complex traits. A major example is single-cell transcriptomics, enabling the accurate characterization of cell-specific expression and interaction and providing an additional layer in gene–trait associations diluted in bulk transcriptomes [10]. Recent single-cell *cis*-eQTL studies have also validated that the transcriptional orchestration often acts in a cell-specific manner [7]. Integrating single-cell *cis*-eQTLs with GWAS through Mendelian Randomization (MR) [11] provides a formal framework to assess the relevance of genetically proxied gene expression in specific cell states. However, systematic applications of single-cell MR to complex inflammatory diseases remain limited, and the extent to which single-cell-derived associations are captured by conventional bulk approaches is unclear.

**Figure 1:**
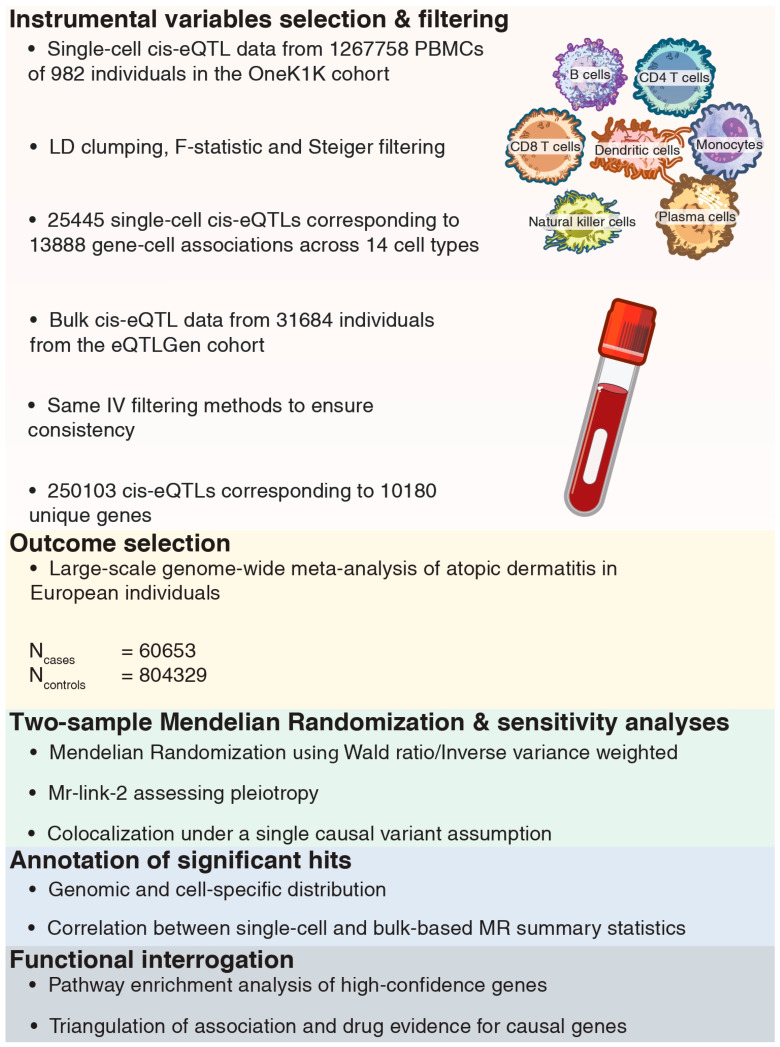
The flowchart of our study.

To characterize the genomic context of AD at the single-cell level, we integrated *cis*-eQTLs across 14 immune cell types from the OneK1K cohort [7] with GWAS summary statistics for AD [2] using a two-sample MR framework [11]. We described the genomic and cellular distribution of significant signals. Through multi-step filtering criteria, we refined our list of candidate risk genes. We compared the magnitude of effect and intersection of single-cell MR results with bulk-based methods from the eQTLGen consortium [12]. An annotation of the high-confidence genes was conducted using complementary functional and pharmacological resources. Our integrative approach, as shown in Figure 1, provides a fine-grained genetic architecture at an immune cell-specific layer, highlighting the added value of single-cell resolution for gene prioritization and translational relevance in AD.

## 2. Results

### 2.1. Genomic Architecture of Single-Cell Associations in Atopic Dermatitis

We analyzed independent *cis*-eQTLs associated with cell-specific transcriptional regulation across 14 different immune cell types from 982 participants in the OneK1K cohort [7]. Variants associated with gene expression at a nominal *p*-value ≤ 1 × 10^−5^ were retained, corresponding to a false discovery rate (FDR)-adjusted threshold of 0.05 [13]. Instrumental variables (IVs) were constructed through linkage disequilibrium (LD) clumping at an r^2^ threshold of 0.1, with correlations estimated using genotype data from 10,000 unrelated participants of European ancestry from the UK Biobank [14]. Additional quality control and filtering steps are detailed in the Section 4.

**Figure 2:**
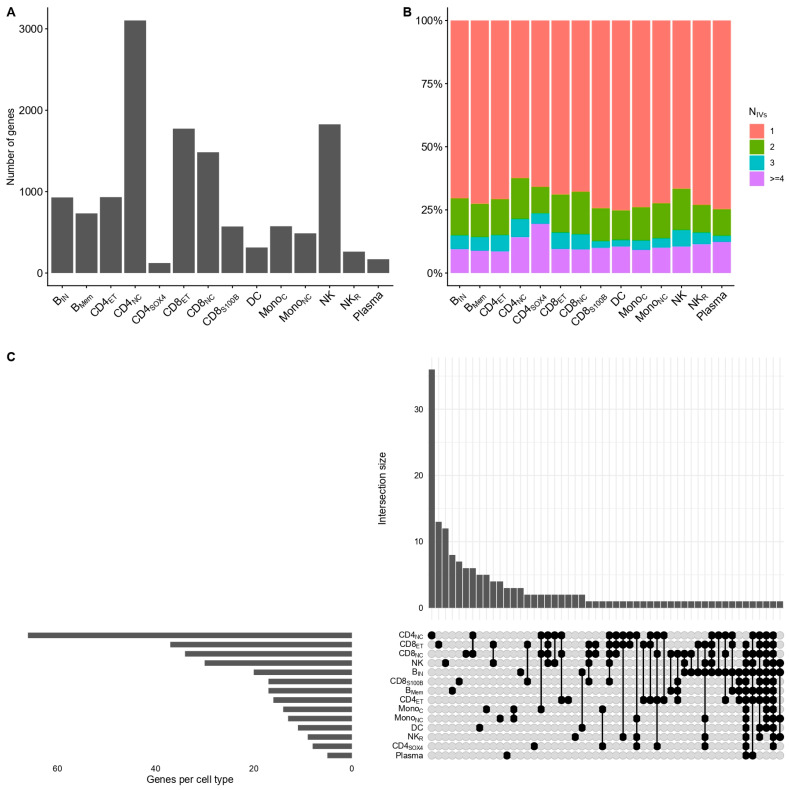
Transcriptional architecture of atopic dermatitis at the single-cell level. (**A**) Number of genes with at least one valid IV per cell type. (**B**) Stacked bar plot reporting the number of IVs available for each gene per cell type. (**C**) UpSet plot illustrating the number of overlapping genes per cell type. Abbreviations: B_IN_, immature and naïve B cells; B_Mem_, memory B cells; CD4_ET_, effector memory CD4 T cells; CD4_NC_, naïve and central memory CD4 T cells; CD4_SOX4_, CD4 T cells expressing SOX4; CD8_ET_, effector memory CD8+ T cells; CD8_NC_, naïve and central memory CD8 T cells; CD8_S100B_, CD8 T cells with expression of S100B; DC, dendritic cells; Mono_C_, classical monocytes; Mono_NC_, non-classical monocytes; NK, natural killer cells; NK_R_, recruiting natural killer cells; Plasma, plasma cells.

After LD clumping and thresholding, 25,445 independent *cis*-acting eQTLs corresponding to 13,288 genes across 14 immune cell types were retained for downstream analyses. The number of testable genes varied across cell states, with naïve/central memory CD4 (CD4_NC_) T cells contributing the largest number (*n* = 3104), followed by natural killer (NK) cells (*n* = 1827), whereas CD4_SOX4_ cells contributed the fewest (*n* = 123; Figure 2A). Consistent with sparse single-cell *cis*-regulatory architectures, most gene–cell combinations were instrumented by a single independent variant (Figure 2B).

We next performed two-sample Mendelian Randomization (MR) using cell-specific *cis*-eQTLs as exposures and GWAS summary statistics for AD as the outcome. MR estimates were obtained using the Wald ratio for one IV and inverse variance weighted (IVW) estimate when two or more IVs were available. At an FDR threshold of ≤0.05, we identified 303 significant MR cell-specific gene–trait associations, corresponding to 164 unique genes across all 14 immune cell types when using the Wald ratio or IVW as the primary estimates (Table S1). This number was reduced to 297 total associations (162 unique genes) through MR sensitivity analyses (Methods). The assessment of the cell specificity of gene–trait associations revealed limited overlap across cell states. Specifically, 108 of the 162 unique genes (66.67%) showed significant effects in a single-cell type (Figure 2C). In contrast, only two genes, including the ribosomal protein *RPS26* and the long non-coding RNA *CUTALP* pseudogene, were identified in 13/14 cell types excluding plasma and CD4_NC_ cell types, respectively (Table S1).

To characterize the architecture of FDR-significant MR genes, we examined the distribution of significant genes across (i) immune cell states and (ii) genome-wide significant (GWS) regions. Although CD4_NC_ T cells harbored the largest number of MR-significant genes (*n* = 66), this cell state also contributed the largest number of testable genes overall (*n* = 3104; Figure 2A). A formal enrichment analysis using Fisher’s exact test did not identify any immune cell type with a significant over-representation of MR-significant genes after FDR correction (Table 1). We further assessed the genomic context of MR-significant genes by mapping them to GWS loci, defined as ±1 MB around each lead variant (Methods). Of the 162 unique genes, 77 (47.53%) were mapped within GWS regions, while 85 (52.46%) were located outside these loci. A similar pattern was observed when conditioning on cell-specific associations (Figure S1). These results indicate that the genetically predicted transcriptional effects in AD are not restricted in GWS loci and are broadly distributed across discrete immune cell states.

### 2.2. Gene Prioritization

To refine our list of prioritized risk genes for AD at the single-cell level, we implemented a three-step procedure. First, a gene was required to show FDR-corrected significance in the primary analysis, using the Wald ratio or IVW, and consistent directionality across all sensitivity analyses. Second, since more than half of the significant associations were estimated using a single IV (*n* = 166, 55.89%), and hence sensitivity analyses were not applicable [15], we employed Mr-link-2 [16], a pleiotropy-robust likelihood function that requires only summary statistics and models the LD between all associated variants (*p*-value ≤ 1 × 10^−5^) in the *cis* region. For Mr-link-2, we required nominal significance for the causal effect estimate $\hat{\alpha }$ (*p*-value ≤ 0.05) and non-significant horizontal pleiotropic variance $\sigma_{y}$ (*p*-value ≥ 0.05). This step identified 59 unique genes across 14 immune cell types, corresponding to 114 unique gene–trait associations. The results for all MR-link-2 gene–trait associations are available in Table S2.

**Figure 3:**
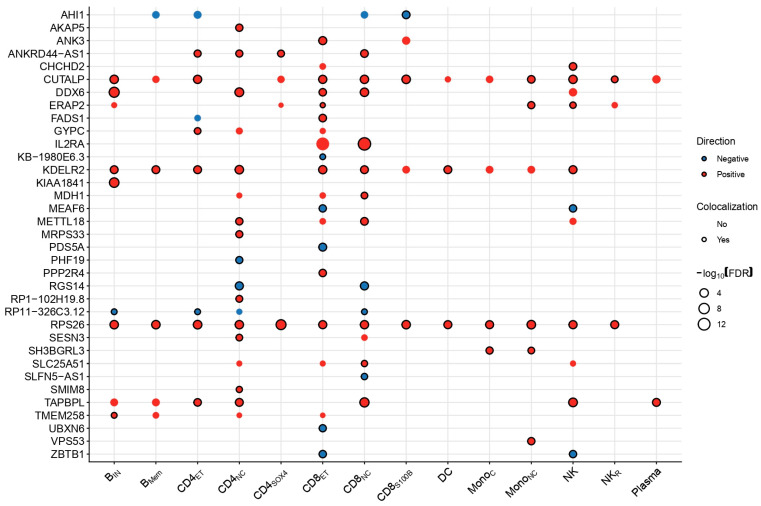
Heatmap summarizing Mendelian Randomization and sensitivity analysis estimates of immune cell-specific expression patterns in AD risk. The outline color of each node represents existence of genetic colocalization between the genetically regulated gene expression of a gene (*y* axis) in a specific cell type (*y* axis) and AD. Gene–trait pairs with posterior probability (PP.H4/(PP.H3 + PP.H4) ≥ 0.6 were declared as colocalized. The fill color of each node represents the signed effect of the cell-specific expression of a gene in AD risk. The size of each node represents the negative base 10 logarithm of the MR association FDR-corrected *p*-value. Abbreviations: B_IN_, immature and naïve B cells; B_Mem_, memory B cells; CD4_ET_, effector memory CD4 T cells; CD4_NC_, naïve and central memory CD4 T cells; CD4_SOX4_, CD4 T cells expressing SOX4; CD8_ET_, effector memory CD8+ T cells; CD8_NC_, naïve and central memory CD8 T cells; CD8_S100B_, CD8 T cells with expression of S100B; DC, dendritic cells; Mono_C_, classical monocytes; Mono_NC_, non-classical monocytes; NK, natural killer cells; NK_R_, recruiting natural killer cells; Plasma, plasma cells; FDR, false discovery rate.

Third, we performed statistical colocalization under a single causal variant assumption, defining colocalization as a posterior probability (PP) of a shared association PP.H4/(PP.H3 + PP.H4) ≥ 0.6. Summary statistics for all colocalization analyses are presented in Table S3. Across all analyses, 80 gene–trait associations remained robust, corresponding to 35 genes across all 14 immune cell subtypes. Sensitivity analyses redefined our prioritized genes and provided additional insights into cell-specific gene–trait associations in AD. For instance, *IL2RA*, a drug target for AD, showed colocalization signals in CD8_NC_ but not in effector memory CD8 (CD8_ET_) T cells, whereas AD-*KDELR2* associations were non-colocalized in monocytes (Figure 3). The only gene–trait association surviving all sensitivity analyses was *RPS26*, demonstrating positive associations with AD across 13/14 cell states excluding plasma cells (Figure 3).

Genetically proxied transcriptional activity of significant genes showed largely consistent directional estimates across implicated cell states. Directionality was generally concordant for genes expressed in multiple cell types, with a single exception observed for *FADS1* in effector memory CD4 (CD4_ET_) and CD8_ET_ T cells, where the former did not survive the colocalization threshold (Figure 3). Prioritized genes were mostly mapped in non-GWS loci (59/80, 73.75%) even when conditioning on the cell type (Figure S2) consistently with the FDR-significant genes (Figure S1). Lastly, although CD4_NC_, CD8_NC_ and CD8_ET_ cells harbored the largest number of significant genes (*n* = 13), enrichment analysis with Fisher’s exact test did not support an over-representation of prioritized genes across cell types (Figure S3).

### 2.3. Comparison with Bulk cis-eQTLs

To compare single-cell results with conventional bulk approaches, we performed two-sample MR using *cis*-eQTLs from the eQTLGen consortium [12] as exposure. LD clumping, instrument selection and filtering criteria were matched to the single-cell two-sample MR for consistency. In eQTLGen, we identified 250,103 independent *cis*-eQTLs across 10,180 genes. In total, 711 gene–trait associations reached FDR ≤ 0.05, of which 672 remained significant after sensitivity analyses, including MR-Egger intercept [15] and MR-PRESSO (Methods) [17]. The complete results from the bulk MR analyses are provided in Table S4.

**Figure 4:**
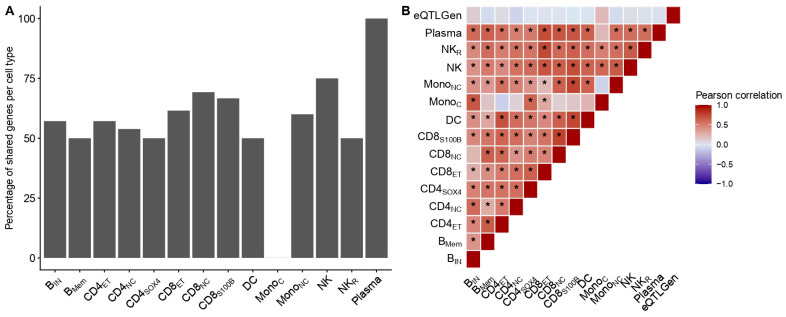
Comparison of single-cell Mendelian Randomization estimates from the OneK1K cohort with bulk Mendelian Randomization estimates from the eQTLGen consortium. (**A**) Bar plot showing the percentage of significant genes (*y* axis) after the intersection of single-cell with bulk Mendelian Randomization results per cell type (*x* axis). (**B**) Pearson correlation matrix between the effects of single-cell and bulk Mendelian Randomization estimates. Asterisk (*) corresponds to FDR-corrected *p*-value ≤ 0.05. Abbreviations: B_IN_, immature and naïve B cells; B_Mem_, memory B cells; CD4_ET_, effector memory CD4 T cells; CD4_NC_, naïve and central memory CD4 T cells; CD4_SOX4_, CD4 T cells expressing SOX4; CD8_ET_, effector memory CD8+ T cells; CD8_NC_, naïve and central memory CD8 T cells; CD8_S100B_, CD8 T cells with expression of S100B; DC, dendritic cells; Mono_C_, classical monocytes; Mono_NC_, non-classical monocytes; NK, natural killer cells; NK_R_, recruiting natural killer cells; Plasma, plasma cells.

Among the 35 genes prioritized through single-cell MR and sensitivity filtering, 22 (62.85%) were also significant using bulk whole blood *cis*-eQTLs, with a largely concordant direction of effects (Figure S4). When stratified by immune cell state, gene–trait associations in classical monocytes (Mono_C_) were absent from the intersection with eQTLGen summary statistics (Figure 4A). Contrastingly, the highest proportion of single-cell prioritized genes recovered in bulk analyses was observed for plasma cells, with *TAPBPL* being validated in both cell-specific single-cell *cis*-eQTL data in plasma cells (beta, 95% confidence intervals (CI): 0.12, 0.07–0.18) and in eQTLGen (beta, 95% CI: 0.045, 0.032–0.058; Figure 4A). Genes identified exclusively at the single-cell level were not detected in bulk MR (Table S1). Moreover, pairwise correlation estimates between effect size estimates from single-cell and bulk *cis*-eQTL MR estimates were low and non-significant (Figure 4B), consistent with the attenuation of cell-specific transcriptional effects in bulk expression models.

### 2.4. Functional Annotation of High-Confidence Genes

Using four complementary layers of genetic evidence, we identified 22 high-confidence genes in AD, supported by both single-cell and bulk *cis*-eQTLs (Figure S5). Through functional enrichment analysis, we found (i) inadequate evidence for the involvement in similar biological pathways (Figure S6) and (ii) limited protein–protein interactions between them even at relaxed interaction thresholds (*p*-value = 0.625; Figure S7). Given the lack of pathway-level convergence, we sought to contextualize prioritized genes individually by employing additional public resources. Disease annotation from the Open Targets platform [18] identified 16 of the 22 prioritized genes with reported associations to AD-related phenotypes, spanning dermatological, immune and allergic contexts, with the largest association scores observed for *IL2RA*, *AHI1* and *FADS1* in asthma (Table S5). The annotation of rodent knockout phenotypes from the Mouse Genome Informatics (MGI) database [19] revealed that only a small subset of genes exhibited immune-related phenotypes including *IL2RA*, whereas most genes were associated with developmental or metabolic traits. Notably, mouse orthologs of *PDS5A* and *CHCHD2* were associated with abnormal skin morphology (Table S6). The assessment of pharmacological tractability with the Pharos database [20] identified *IL2RA* as the only clinically established drug target (Tclin), with *PTPA*, *ERAP2*, *MDH1* and *FADS1* classified as proteins known to bind small molecules with increased efficacy (Tchem), whereas most genes were categorized as having known biological functions (Tbio; 10/22) or proteins of unknown function (Tdark; 3/22) (Table S7). The Querying Drug–Gene Interactions database (DGIdb) [21] identified approved drug–gene interactions for *IL2RA* and *ANK3*, alongside non-approved interactions for *PTPA*, *ERAP2* and *TMEM258* (Table S8). Several identified drugs have been evaluated in clinical trials of AD (data from clinicaltrials.gov; accessed 19 January 2026), including melatonin interacting with *ANK3* for sleep disturbances in children with AD (NCT: NCT01638234) [22], as well as agents interacting with *IL2RA* such as the antihistamine cetirizine (NCT: NCT00375713), mercaptopurine (NCT: NCT05969730) [23] and prednisolone (NCT: NCT00445081) [24] compared to cyclosporine, another widely employed pharmacotherapy for skin diseases including psoriasis [25].

## 3. Discussion

In this study, we evaluated the genetically mediated effects of 13,288 gene–immune cell associations on AD by leveraging single-cell *cis*-eQTL data from 14 discrete immune cell types [7]. Peripheral blood immune populations represent a central component of systemic immune dysregulation in AD. The single-cell *cis*-eQTL from the OneK1K cohort captures major circulating immune lineages implicated in allergic inflammation [7], thus enabling cell-specific regulatory effects that cannot be captured in bulk models. Using a conventional two-sample MR framework, we characterized the distribution of transcriptome-wide associations at the single-cell level. Although most gene–trait associations (66.67%) reported a cell-specific effect, the enrichment analysis did not support the over-representation of a specific cell type in AD (Table 1). Comparing our approach to bulk *cis*-eQTL results with a much larger sample size (*n* = 31,684 in eQTLGen compared to *n* = 982 in OneK1K) revealed a poor correlation between single-cell and bulk MR effect estimates, supporting the attenuation of cell-specific regulatory signals in bulk transcriptomes. Triangulating functional and drug–gene interaction evidence revealed 22 relatively independent-acting genes with genetically mediated effects in AD. Collectively, our results corroborate a polygenic architecture in which genetically proxied transcriptional effects act in a context- and cell-specific manner rather than through secluded pathways.

A central observation was the limited overlap of MR-significant genes across immune cell types. Nearly two-thirds of prioritized genes were detected in a single-cell state (Figure 2C), and formal enrichment analyses did not support the over-representation of associations in any specific immune compartment (Table 1). This pattern suggests that genetically proxied transcriptional effects in AD are distributed across multiple immune cell states rather than concentrated within a dominant lineage, although power differences and sparse *cis*-eQTL architectures in single-cell data might limit detectability. This observation complements our previous work demonstrating a dense genomic architecture of transcriptional regulation in AD [9] at the bulk level. Contrastingly, our current approach further enabled us to delineate the known immunological complexity in AD by leveraging single-cell *cis*-eQTL data, highlighting distinct contributions and validating the multitude of cell types participating in the pathogenesis of AD with distinct functional roles.

While more than 300 MR-significant associations were initially detected, only a small subset survived pleiotropy-robust modeling and colocalization (Figure 3). The effect directionality was largely consistent across cell states for the genes detected in multiple cell types when comparing all results (Figure 4B) and conditioning on significance (Figure 3), indicating a stable genetic regulation in AD despite the cellular context. This is also of relevance when intersecting significant single-cell MR results with bulk MR using the eQTLGen data, where approximately two-thirds of single-cell prioritized genes were also detected, with a consistent direction of effect (Figure S4). However, cell-restricted associations, as in the case with Mono_C_, were not recovered in bulk analyses (Figure 4A). This demonstrates the improved ability of single-cell *cis*-eQTLs to detect context-specific associations not captured in bulk analyses without a necessarily high correlation of effect estimates (Figure 4B).

Stringent sensitivity filtering further refined the set of candidate genes. The functional enrichment of the 22 high-confidence genes supported by both single-cell and bulk evidence revealed a limited convergence at the pathway or network level. Neither functional enrichment nor protein–protein interaction analyses identified significant clustering, even under relaxed thresholds. The inference of the independent mechanism of action using multi-modal genetic and functional evidence prompted us to investigate their roles individually. Using external annotations provided a biological context for most genes, identifying skin and/or immune-related gene–trait associations in 16/22 genes from the Open Targets platform (Table S6). Some of these genes have been previously identified as risk genes for AD [2,9], with the examples of *RGS14* exerting its role in CD4_NC_ and CD8_NC_ T cells, pleiotropic *RPS26* participating in 13/14 cell states, and *IL2RA*. The latter gene also showed evidence for druggability in AD, where a single clinical trial evaluated the efficacy of the T-regulatory (Treg)-specific *IL2RA* agonist rezpegaldesleukin independently of peripheral bulk CD45^+^CD3^+^CD8^+^ T cell populations (NCT: NCT04081350) [26]. This pharmacologic expansion of Tregs occurred in a dose-dependent manner against a cytokine milieu supporting CD8 T cell survival, including IL15 and CXCL16 [27], potentially attenuating pathogenic CD8 sub-phenotypes indirectly. In our study, we showed that the genetically proxied increased expression of *IL2RA* in CD8_NC_ T cells increased AD risk (Figure 3).

Our framework is further able to resolve locus-specific mechanisms, providing novel insights into how genetic variants may affect different genes at different cell states and immune contexts. Specifically, we observed two transcriptome-wide significant associations at the 11q12.2 chromosomal region encompassing *TMEM258* and *FADS1* genes, among others, an established risk locus [2] for AD with pleiotropic associations extending beyond dermatological traits such as neuropsychiatric disorders [5]. Our study suggests discrete roles for both genes in different immune cells, where the genetically proxied expression of *FADS1* was colocalized and associated exclusively in CD8_ET_ T cells, whereas *TMEM258* showed robust associations in immature and naive B (B_IN_) cells (Figure 3). Similarly, the genetically regulated reduced expression of the T cell-associated epigenetic regulator *PHF19* in CD4_NC_ T cells showed inverse associations with AD risk, while *CUTALP* reported no signal in CD4_NC_ cells and was associated with diverse cell types including Mono_NC_, NK cells and B_IN_ (Figure 3). In the same 9q33.2 chromosomal region, we have previously highlighted another gene, *TRAF1*, associated with AD in bulk-level analyses [5]. We also identified disease-relevant genes including *ERAP2* and *TAPBPL*, potentially shaping T cell activation and NK cell responsiveness through shared modulatory signals in CD4_NC_ and NK cells (Figure 3), and *ANK3* in CD4_NC_ T cells, a gene mostly associated with neurodevelopmental disorders and previously linked with AD in keratinocytes [28].

Several limitations merit consideration. Single-cell *cis*-eQTL sample sizes remain modest relative to bulk resources, limiting the power for some cell states and favoring sparse instrument architectures. This limitation may contribute to the under-detection of shared regulatory effects and increased variance of effect estimates. To mitigate the risk for potential false-positive rates, we employed sensitivity analyses including Steiger filtering, MR-link-2 as a pleiotropy-robust method, which is of relevance in cases when only a single IV was used, and genetic colocalization to derive a list of high-confidence risk genes for AD. In addition, despite our genes surviving multiple sensitivity analyses, functional annotation resources remain incomplete for many cases, constraining biological interpretation and potential therapeutic avenues. Nonetheless, the consistency of findings across multiple analytical layers supports the robustness of the core observations, while our study further prioritized some genes for functional follow-up. Moreover, our estimates are primarily derived from individuals of European ancestry, obscuring the generalizability of the findings to other ethnicities. Lastly, our approach was limited to peripheral blood cells and not other AD-relevant tissues, where a significant amount of AD-associated variants may exert their effects in other tissues, such as skin, which also includes immune cells, though with different stimuli.

In summary, our results demonstrate that genetically mediated transcriptional effects in AD are highly cell-specific and only partially captured by bulk transcriptomic approaches. By integrating single-cell *cis*-eQTLs with MR and sensitivity analyses, we delineate a refined set of candidate genes that reflect the polygenic and context-dependent nature of immune regulation in AD. More broadly, these findings support a model of immune-mediated disease risk in which genetic effects are expressed through cell-specific transcriptional programs. These findings provide a framework for future functional studies and illustrate the value of cellular resolution for gene prioritization in complex, immune-mediated diseases.

## 4. Materials and Methods

### 4.1. Data Sources

All analyses were conducted on the GRCh37/hg19 human genome version. Summary-level single-cell *cis*-eQTL data were retrieved from the OneK1K cohort, which generated data from 1,267,758 peripheral blood mononuclear cells in 982 healthy European individuals [7]. Based on the single-cell data, the OneK1K cohort identified 14 different immune profiles including B_IN_, memory B cells (B_Mem_), CD4_ET_, CD4_NC_, CD4 T cells expressing SOX4 (CD4_SOX4_), CD8_ET_, CD8_NC_, CD8 T cells expressing S100B (CD8_S100B_), DC, Mono_C_, non-classical monocytes (Mono_NC_) NK, recruiting NK cells (NK_R_) and plasma cells. For OneK1K, we transformed the nonparametric association statistic Spearman’s correlation $\rho_{i}$ for each variant i to standardized allelic effect size $\beta_{i}$ as $\beta_{i}=\frac{\rho_{i}}{\sqrt{2(1-f_{i})}}$, and standard error ${SE}_{i}$ as ${SE}_{i}=\left|\frac{\beta_{i}}{Z_{i}}\right|$, where $Z_{i}=\Phi^{-1}\left(1-\frac{{P-value}_{i}}{2}\right)$, as described elsewhere [29]. Summary-level bulk *cis*-eQTLs were retrieved from the eQTLGen consortium, comprising whole blood transcriptome samples from 31,684 individuals of mostly European ancestry. For the outcome, we selected European GWAS summary statistics from a recent meta-analysis [2] including 864,982 European participants (n_cases_ = 60,653).

To increase the robustness of our LD estimations, we constructed an LD reference panel from 10,000 randomly selected participants of White British ancestry from the UK Biobank [14]. Genotyping of all individuals included in the UK Biobank was performed with the UKB Axiom Affymetrix array as described elsewhere [30]. We followed a conventional approach for construction of the LD reference panel, such as exclusion of third-degree relatives (kinship coefficient > 0.0884), sex discordance, genotype missingness and outlier heterozygosity. We kept variants with minor allele frequency (MAF) ≥ 0.0001.

### 4.2. Selection of Instrumental Variables

We followed the same procedure for both OneK1K and eQTLGen summary data to facilitate accurate comparisons. MR holds three core assumptions, including (i) a strong association with the exposure, (ii) the independence of the IV to pleiotropic associations and (iii) the effect of the IV only through the exposure and not via independent pathways [11]. To satisfy the first assumption, we first defined *cis*-eQTLs as variants within ±1 Mb of the gene region. We applied a soft thresholding of *p*-value ≤ 10^−5^ to identify genes with at least one significant variant. This threshold has been previously applied to *cis*-Mendelian Randomization studies, corresponding to an FDR ≤ 0.05 [13]. Next, we clumped all variants to select independent IVs using an r^2^ threshold of 0.1. We only included variants with F-statistic ≥ 10 to minimize weak instrument bias [31].

### 4.3. Mendelian Randomization

We conducted a two-sample MR approach to ascertain evidence for genetically mediated effects between the cell-specific genetic regulation of a gene on AD using the TwoSampleMR R package v0.6.2. To ensure that the comparisons referred to the same allele, we harmonized the exposure and outcome dataset and excluded palindromic variants. As a last IV selecting approach, we applied Steiger filtering to minimize reverse causality between the *cis*-eQTL and the outcome [32]. Variants explaining more variance in the outcome than the exposure were excluded from MR analyses. In cases where a single IV was available to proxy the expression of a gene in a cell type, we utilized the Wald ratio to estimate their effect on AD. If more than one IV was available, we conducted the IVW method. If the number of IVs was ≥ 3, we conducted sensitivity analyses including the weighted median [33] and MR-Egger [15]. Horizontal pleiotropy was assessed via the intercept term of the MR-Egger regression [15]. If the number of IVs exceeded 4, we further conducted MR pleiotropy residual sum and outlier (MR-PRESSO) sensitivity analysis [17]. All *p*-values were corrected for multiple comparisons with the FDR correction.

As described previously, most gene–cell combinations had a single IV available (Figure 2B) and hence sensitivity analyses could not be conducted. To satisfy all MR assumptions across all analyses, we employed Mr-link-2 to estimate the extent of pleiotropy in the identified genes. Briefly, Mr-link-2 utilizes LD reference panels and pre-harmonized summary statistics from both exposure and outcome associations to estimate (i) the causal effect estimate $\hat{\alpha }$ and (ii) the remaining pleiotropic variance $\sigma_{y}$, which otherwise violates the core assumptions of MR. The explicit modeling of the pleiotropic variance in MR-link-2 enables robustness to LD-induced pleiotropy, especially when inference lies on a single associated locus as is the case with our *cis*-MR study [16]. For Mr-link-2, we allowed computations in the human leukocyte antigen (HLA) region using the --no_exclude_hla option. The same r^2^ threshold for LD computations and genomic window were used as in the primary MR methods.

To assess concordance between cell-specific and bulk transcriptome-wide MR results, we estimated pairwise Pearson correlation coefficients between MR effect size estimates for genes shared between each single-cell immune cell type and bulk eQTLGen analyses. *p*-values of the Pearson correlation estimates were adjusted for multiple comparisons using the FDR method. To account for differences in the number of testable genes across immune cell types, we assessed whether MR-significant genes were over-represented within specific cell states using Fisher’s exact test. For each cell type, a 2 × 2 contingency table was constructed comparing the number of significant genes versus non-significant genes within that cell type to all remaining genes tested in other cell types. *p*-values of Fisher’s exact test were adjusted for multiple comparisons with the FDR method.

### 4.4. Genetic Colocalization

We finally performed genetic colocalization to distinguish between LD and shared causal variants only in the single-cell *cis*-eQTL MR analyses [34]. There are 5 distinct hypotheses in colocalization, including the absence of association of a variant in both traits (H0), association of a variant only for a single trait (H1), association of a variant only for the other trait (H2), association for both traits in the same region but with distinct causal variants (H3), and association for both traits in the same region with the same causal variant (H4). Colocalization was conducted under a single causal variant assumption using the coloc R package v5.2.3 with the default prior probabilities. We declared significant colocalization as PP.H4/(PP.H3 + PP.H4) ≥ 0.6.

A gene–trait association in our single-cell MR analyses was declared significant if it validated our three-step procedure. First, a gene should show FDR ≤ 0.05 at the primary Wald ratio when using a single IV or at the primary IVW estimate when using two or more IVs. Whenever applicable, a gene should show nominal significance (unadjusted *p*-value ≤ 0.05) and a concordant directionality for the effect estimate in sensitivity analyses, including the weighted median, MR-Egger, and MR-PRESSO, while the MR-Egger intercept should not be nominally significant (*p*-value ≥ 0.05). The same criteria were applied for the bulk-based eQTLGen MR analyses. Second, in the Mr-link-2 analysis, a gene–trait association should show nominal significance in the causal effect estimate $\hat{\alpha }$ (*p*-value ≤ 0.05) and a non-significant horizontal pleiotropic variance $\sigma_{y}$ (*p*-value ≥ 0.05). Third, a gene–trait association should show significant colocalization defined as PP.H4/(PP.H3 + PP.H4) ≥ 0.6. To evaluate the genomic distribution of our transcriptome-wide significant hits, we identified lead GWS variants by clumping using a *p*-value threshold of ≤ 5 × 10^−8^, an r^2^ LD threshold of 0.001 and a ±500 kb window. Genes were assigned at GWS loci if their genomic coordinates, as retrieved from the Ensembl database [35], fell within ±1 Mb of a lead variant.

### 4.5. Drug Target Prioritization

We intersected the lists of significant MR results from both single-cell OneK1K and bulk eQTLGen summary statistics to derive a final list of 22 high-confidence genes subjected to follow-up analyses. Specifically, we conducted functional enrichment analysis using Gene Ontology (GO) biological process terms with the g:profiler web-based tool [36], with statistical significance defined as an FDR-adjusted *p*-value ≤ 0.05. To assess the functional interactions between prioritized genes, we submitted our gene list to the Search Tool for the Retrieval of Interacting Genes (STRING) database v12.0 to construct protein–protein interactions at a relaxed confidence threshold of functional interactions at 0.4 [37]. To contextualize the functional relevance and pleiotropic effects of prioritized genes, we integrated evidence from four different databases with different layers of evidence. Disease associations were retrieved from the Open Targets platform, aggregating genetic, pathway-based and text mining evidence among others to compute a gene–trait association score [18]. Mouse phenotype annotations were obtained from the MGI database, the primary curated resource for functional consequences of gene perturbations in laboratory mouse models [19]. In both databases, we focused on annotations that contained AD-relevant terms such as dermatitis, immune, allergy and asthma. To assess pharmacological tractability, we queried the Pharos knowledgebase to obtain target development level (TDL) classification [20], which summarizes the extent of experimental and clinical characterization of each gene. Drug–gene interactions were retrieved from the DGIdb v5.0 database [21]. For drugs with evidence for interactions with the high-confidence genes, we searched for related drug trial targets on the clinicaltrials.gov platform (access date: 20 January 2026).

## Figures and Tables

**Table 1:** Enrichment analysis of cell-specific effects using Fisher’s exact test.

Cell Type	Significant Genes	Total Genes	Odds Ratio	*p*-Value	FDR
B_IN_	20	929	0.937	0.909	0.979
B_Mem_	17	733	1.015	0.898	0.979
CD4_ET_	16	933	0.735	0.303	0.708
CD4_NC_	66	3104	0.91	0.535	0.851
CD4_SOX4_	8	123	2.895	0.009	0.128
CD8_ET_	37	1774	0.899	0.608	0.851
CD8_NC_	34	1484	1.002	1	1
CD8_S100B_	17	572	1.318	0.254	0.708
DC	11	314	1.552	0.177	0.708
Mono_C_	14	575	1.068	0.774	0.979
Mono_NC_	13	488	1.172	0.537	0.851
NK_R_	9	263	1.512	0.211	0.708
NK	30	1827	0.686	0.051	0.36
Plasma	5	170	1.291	0.599	0.851

Abbreviations: B_IN_, immature and naïve B cells; B_Mem_, memory B cells; CD4_ET_, effector memory CD4 T cells; CD4_NC_, naïve and central memory CD4 T cells; CD4_SOX4_, CD4 T cells expressing SOX4; CD8_ET_, effector memory CD8+ T cells; CD8_NC_, naïve and central memory CD8 T cells; CD8_S100B_, CD8 T cells with expression of S100B; DC, dendritic cells; Mono_C_, classical monocytes; Mono_NC_, non-classical monocytes; NK, natural killer cells; NK_R_, recruiting natural killer cells; Plasma, plasma cells; FDR, false discovery rate.

## Data Availability

UK Biobank data are available to bona fide researchers for health-related research in the public interest through application for access (https://www.ukbiobank.ac.uk/, accessed on 23 February 2026). The data presented in the study are openly available in the GWAS catalog (https://www.ebi.ac.uk/gwas/, accessed on 23 February 2026). Single-cell cis-eQTL data from the OneK1K cohort are available at https://onek1k.org/, accessed on 23 February 2026. Bulk cis-eQTL data from the eQTLGen consortium are available at https://www.eqtlgen.org/, accessed on 23 February 2026. Instrumental variables and code for analysis are available at https://github.com/antonatosc/AD_singlecell-MR, accessed on 23 February 2026.

## Supplementary Materials

The following supporting information can be downloaded at: https://www.mdpi.com/article/10.3390/ijms27052226/s1.
