# Supplementary material for: Single-Cell cis-Mendelian Randomization Reveals Cell-Specific Genetic Mechanisms Underlying Atopic Dermatitis
Source: Int J Mol Sci. 2026 Feb 27;27(5):2226. doi: 10.3390/ijms27052226 (PMC12985171; doi:10.3390/ijms27052226)

Figure S1. Genomic distribution of significant genes using single-cell *cis*-eQTLs.

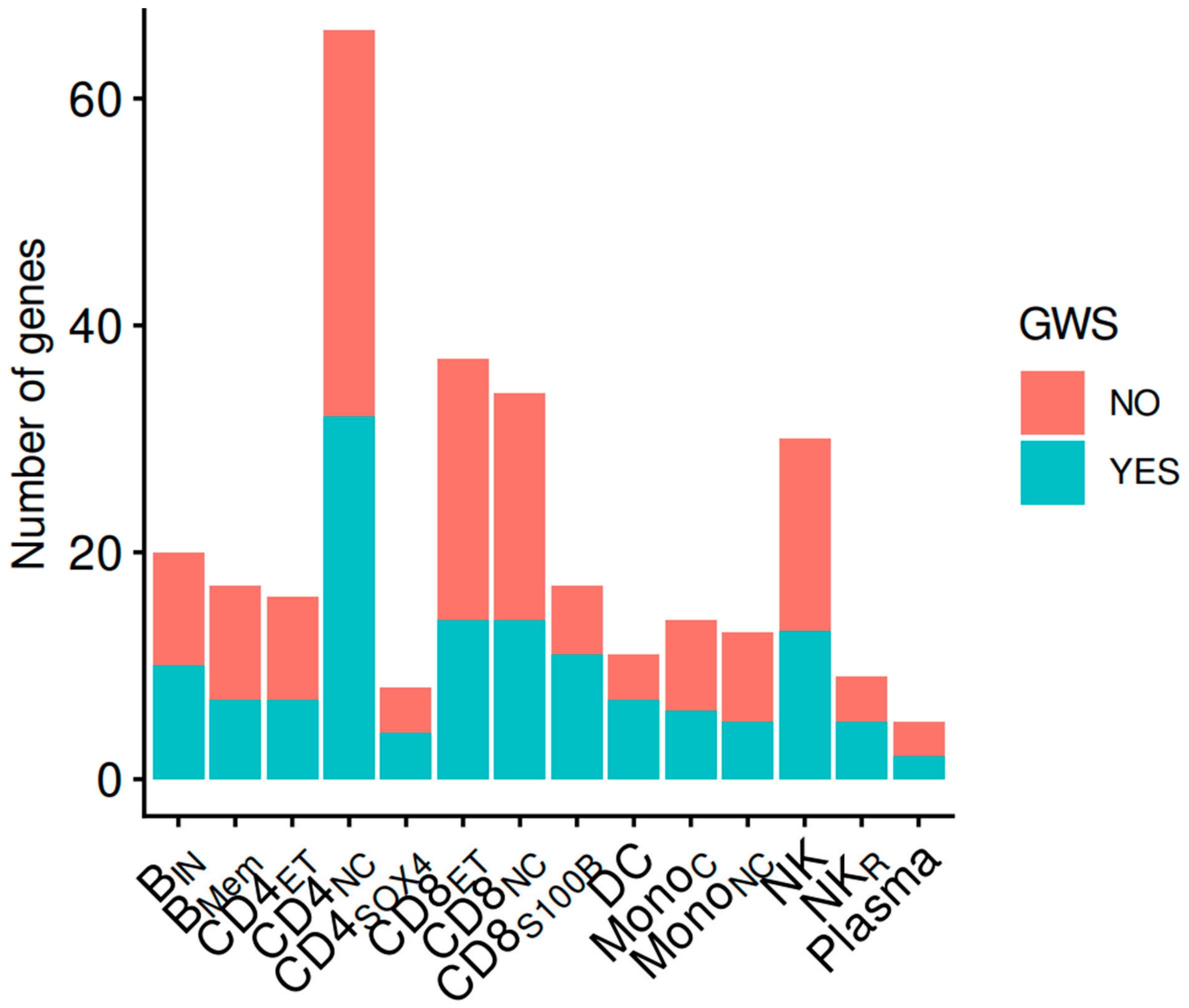

Figure S2. Genomic distribution of significant genes using both single-cell *cis*-eQTLs and bulk *cis*-eQTLs.

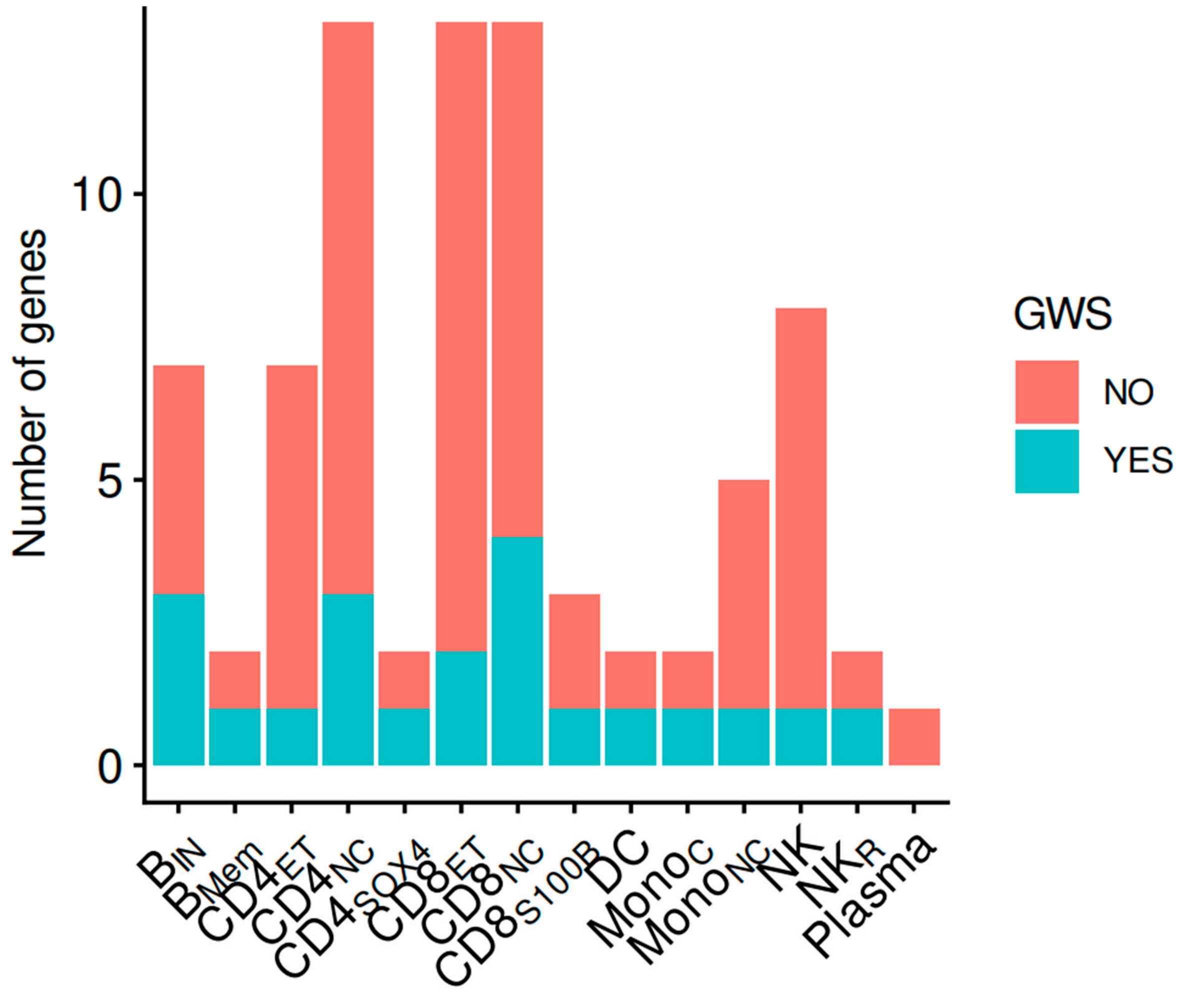

Figure S3. Distribution of significant genes across cell types using single-cell *cis*-eQTLs.

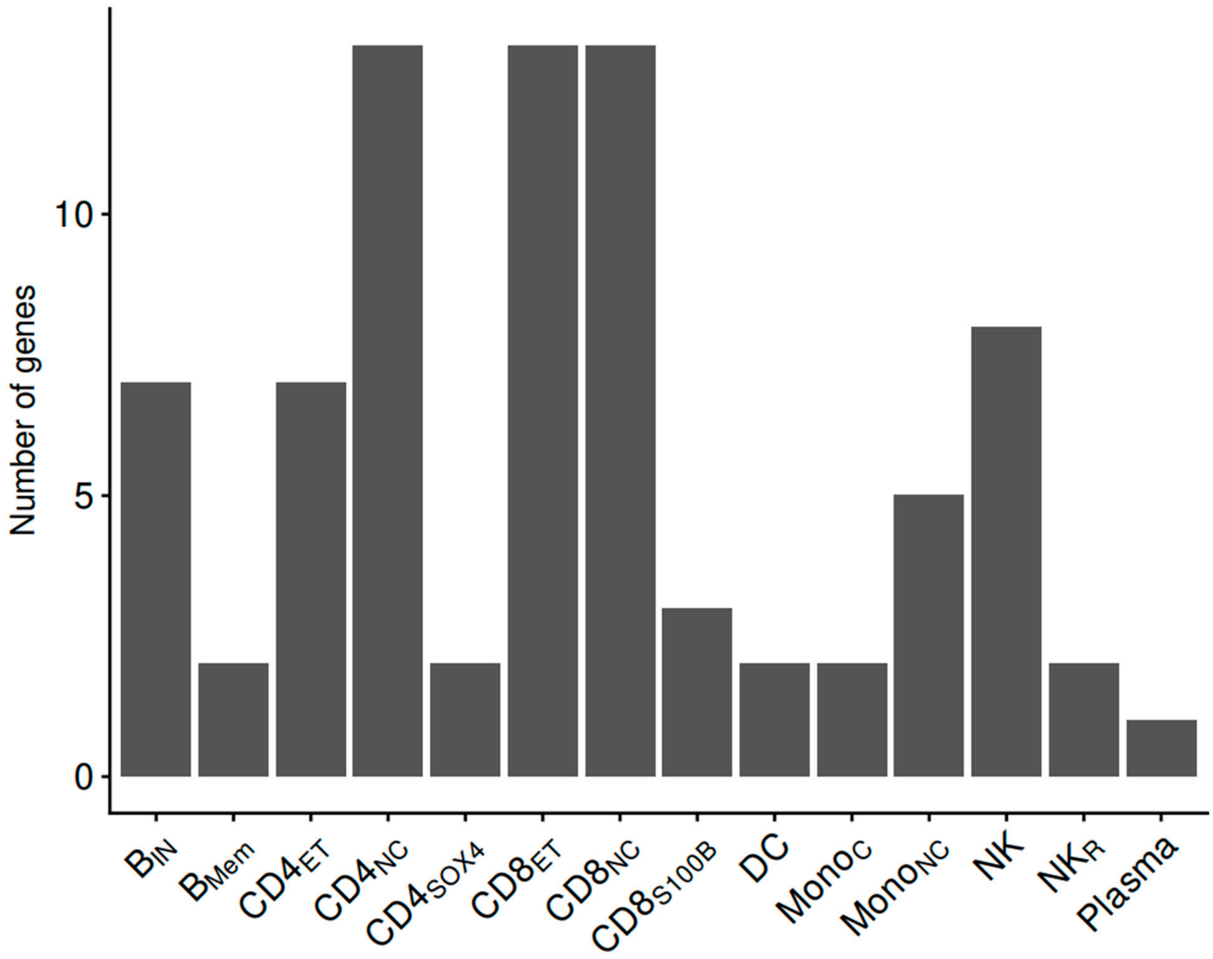

Figure S4. Comparison of effect size estimates for significant genes both single-cell *cis*-eQTLs and bulk *cis*-eQTLs.

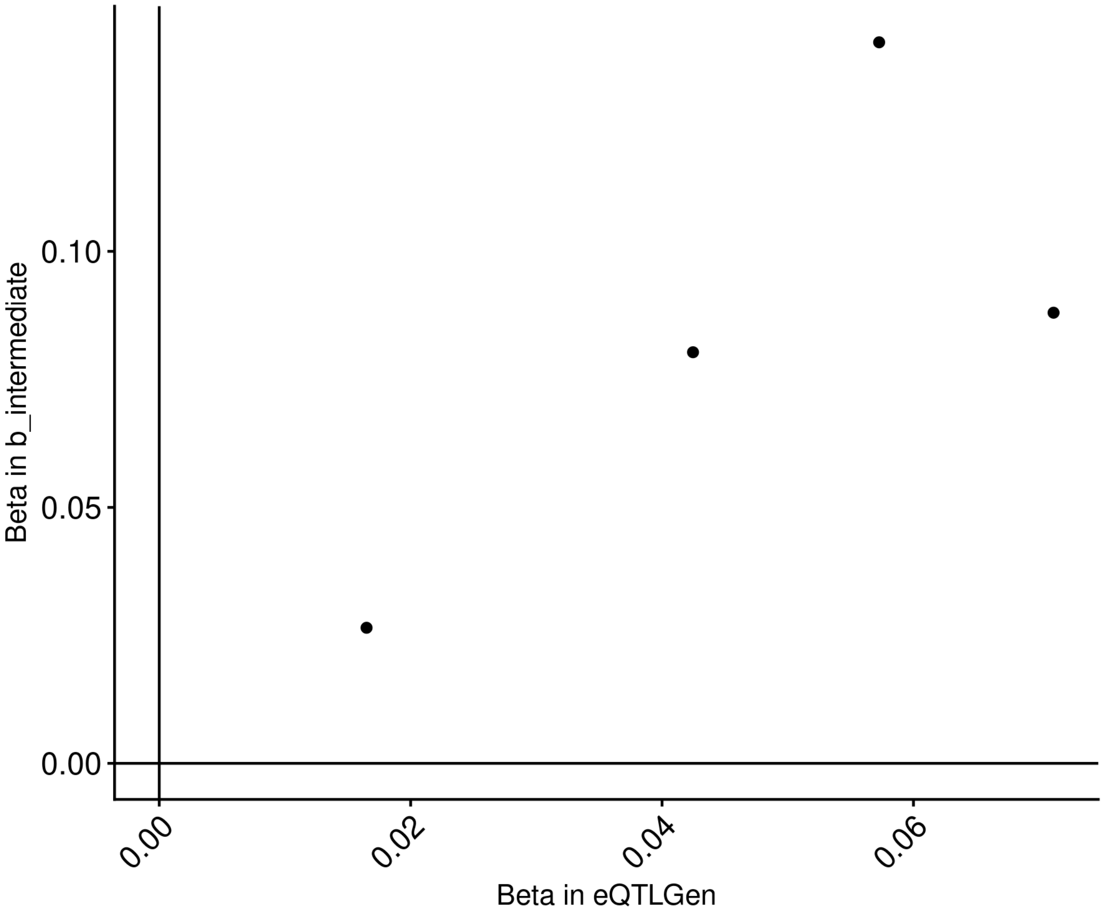

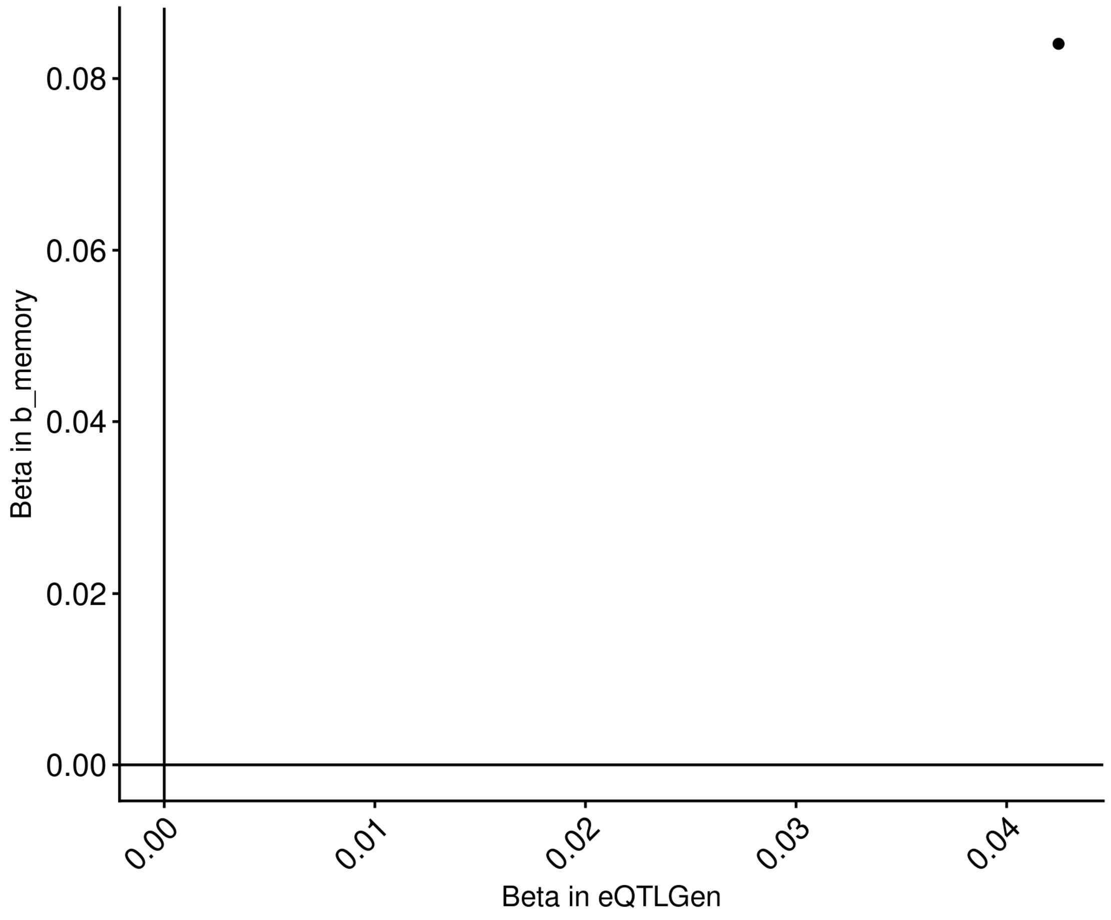
0.08
0.06
0.04
0.02
0.00
Beta in b_memory
0.00
0.01
0.02
0.03
0.04
Beta in eQTLGen

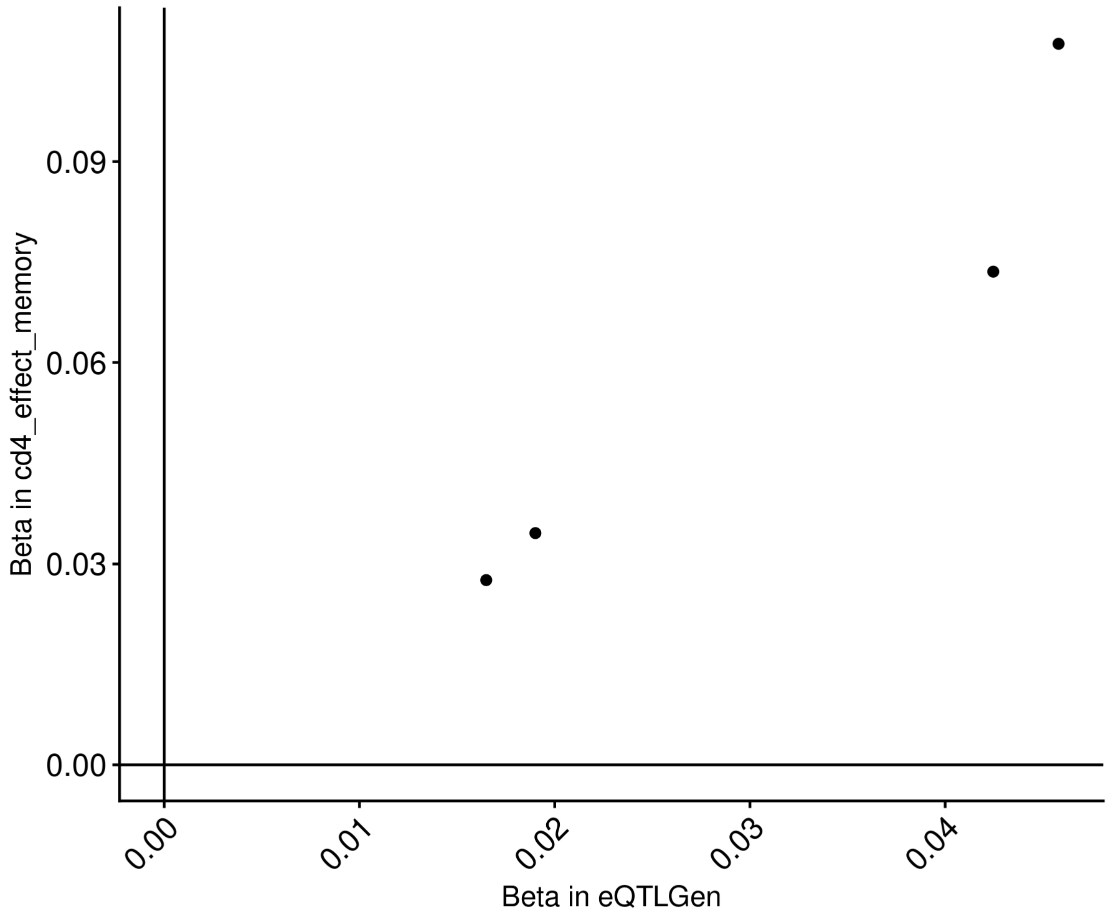
Beta in cd4_effect_memory
0.09
0.06
0.03
0.00
0.00
0.01
0.02
0.03
0.04
Beta in eQTLGen

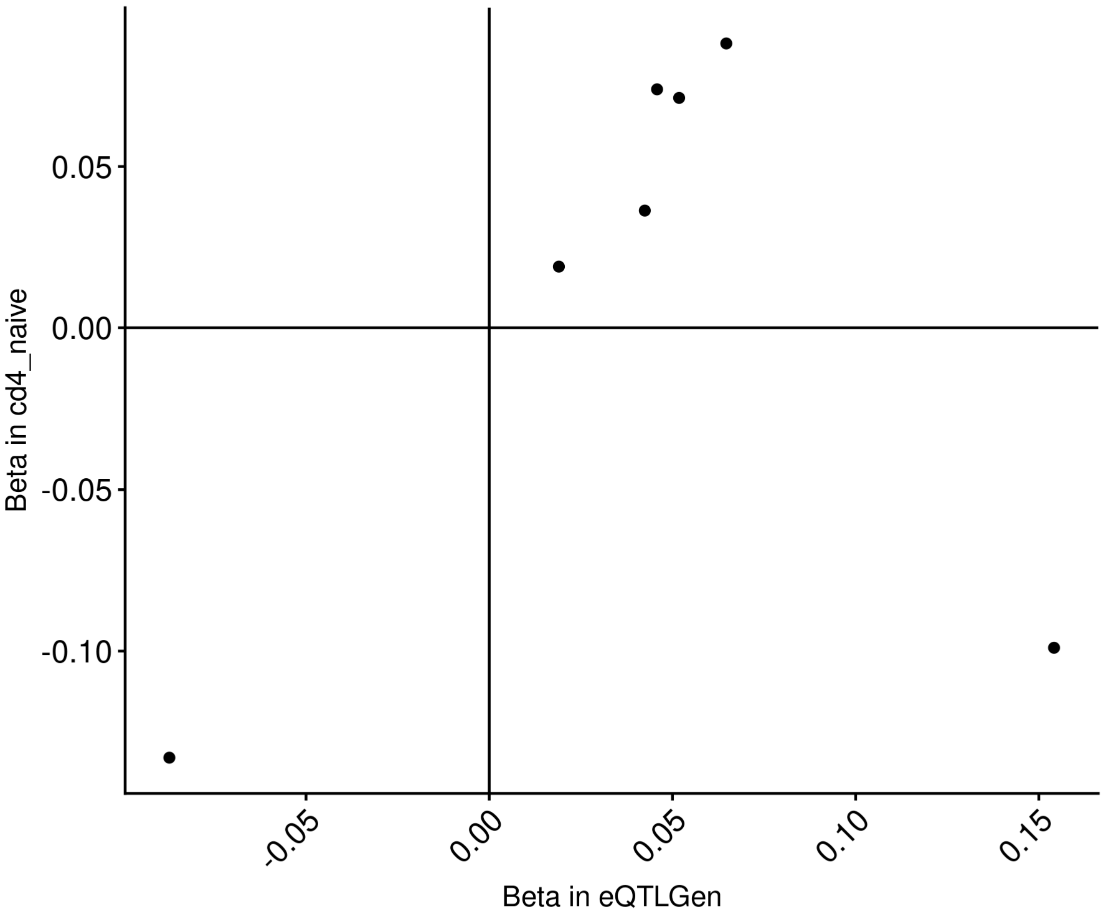
0.05
0.00
-0.05
-0.10
Beta in cd4_naive
-0.05
0.00
0.05
0.10
0.15
Beta in eQTLGen

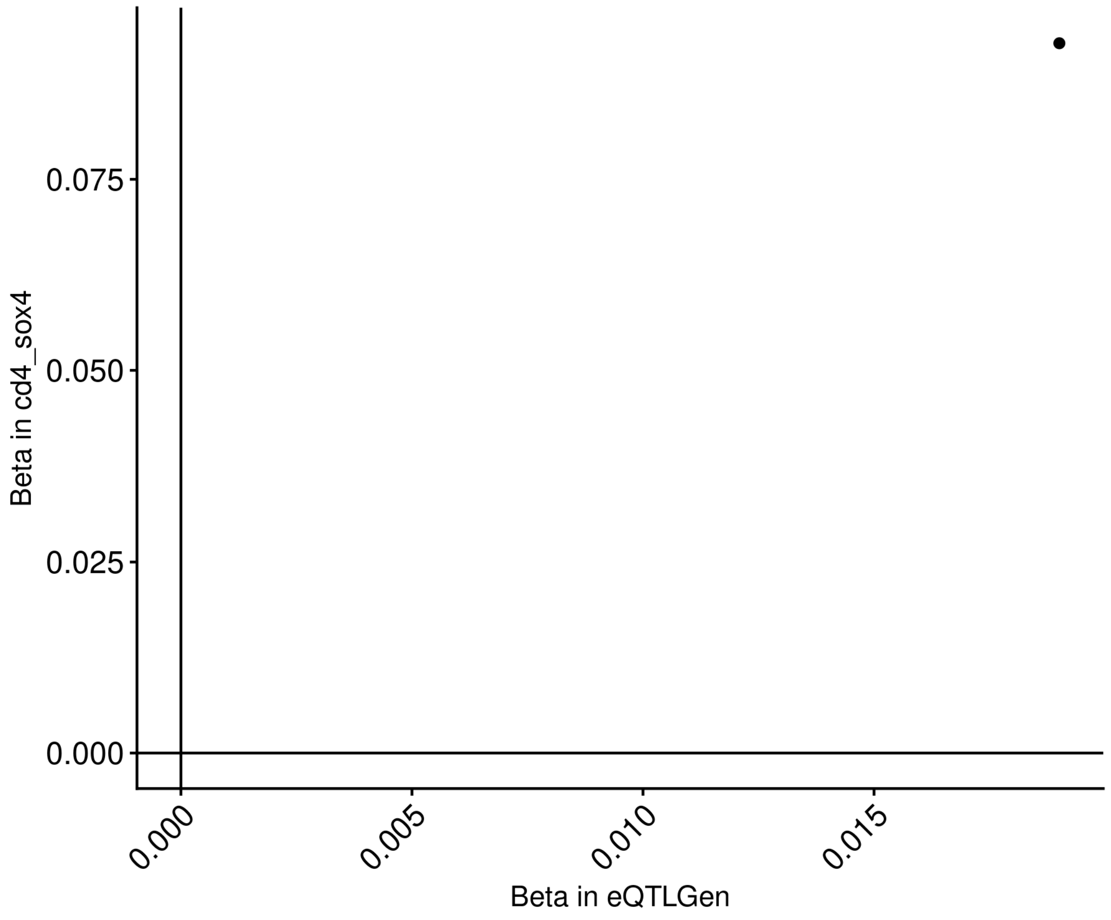

0.075
0.050
0.025
0.000
Beta in cd4_sox4
0.000
0.005
0.010
0.015
Beta in eQTLGen

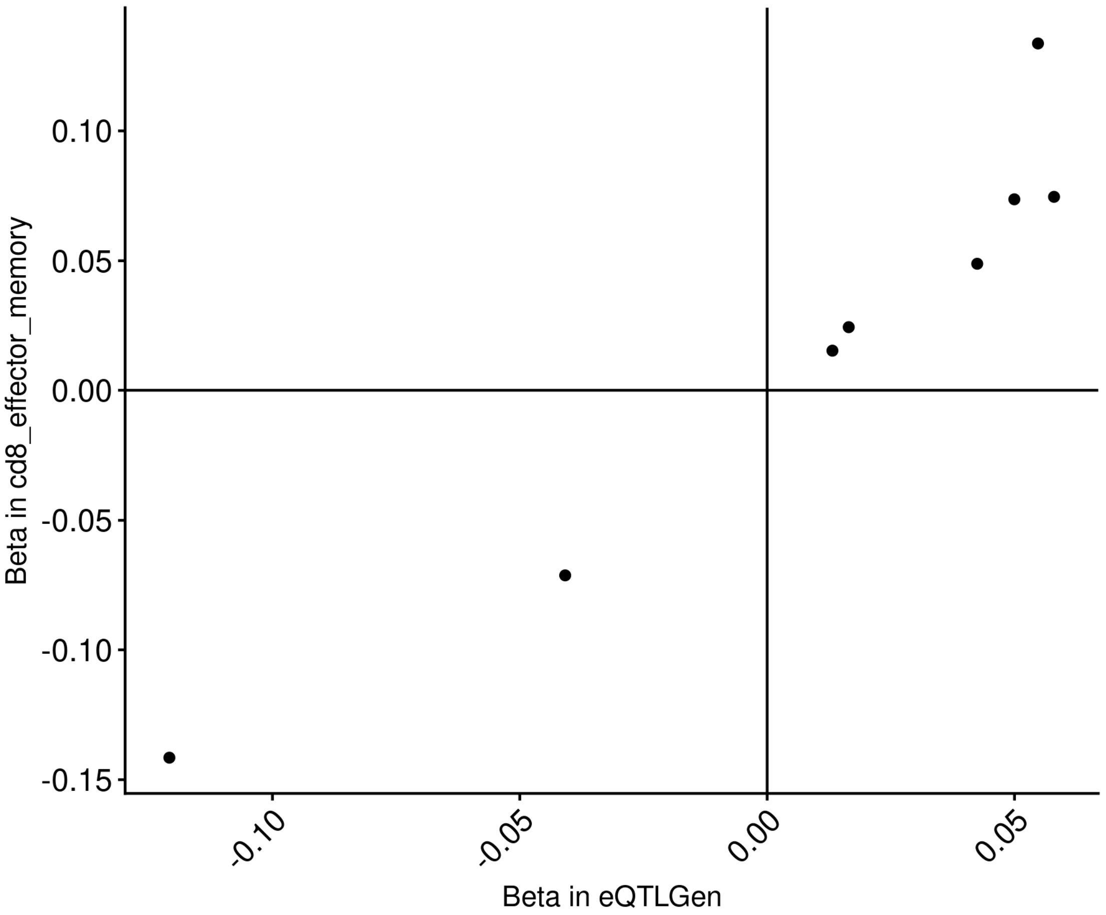

Beta in cd8_effector_memory
0.10
0.05
0.00
-0.05
-0.10
-0.15
-0.10
-0.05
0.00
0.05
Beta in eQTLGen

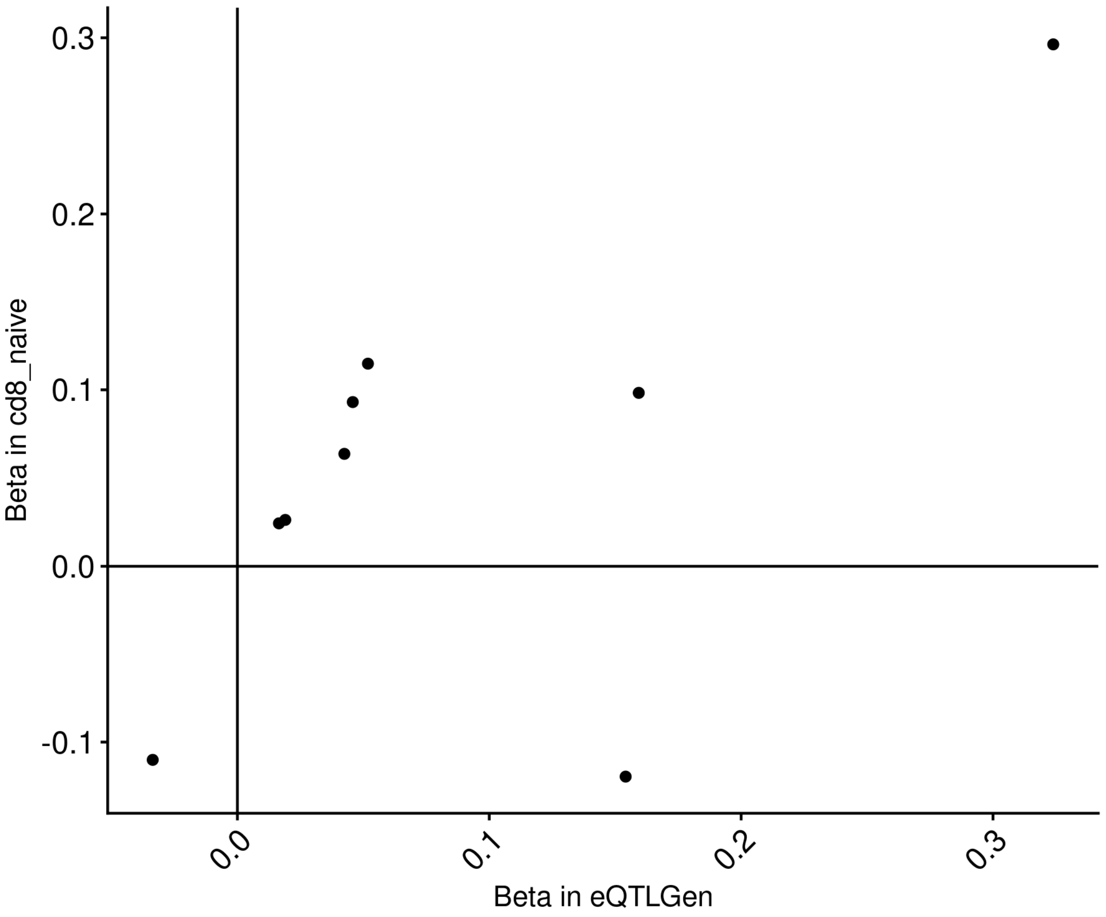

0.3
0.2
0.1
0.0
-0.1
Beta in cd8_naive
0.0
0.1
0.2
0.3
Beta in eQTLGen

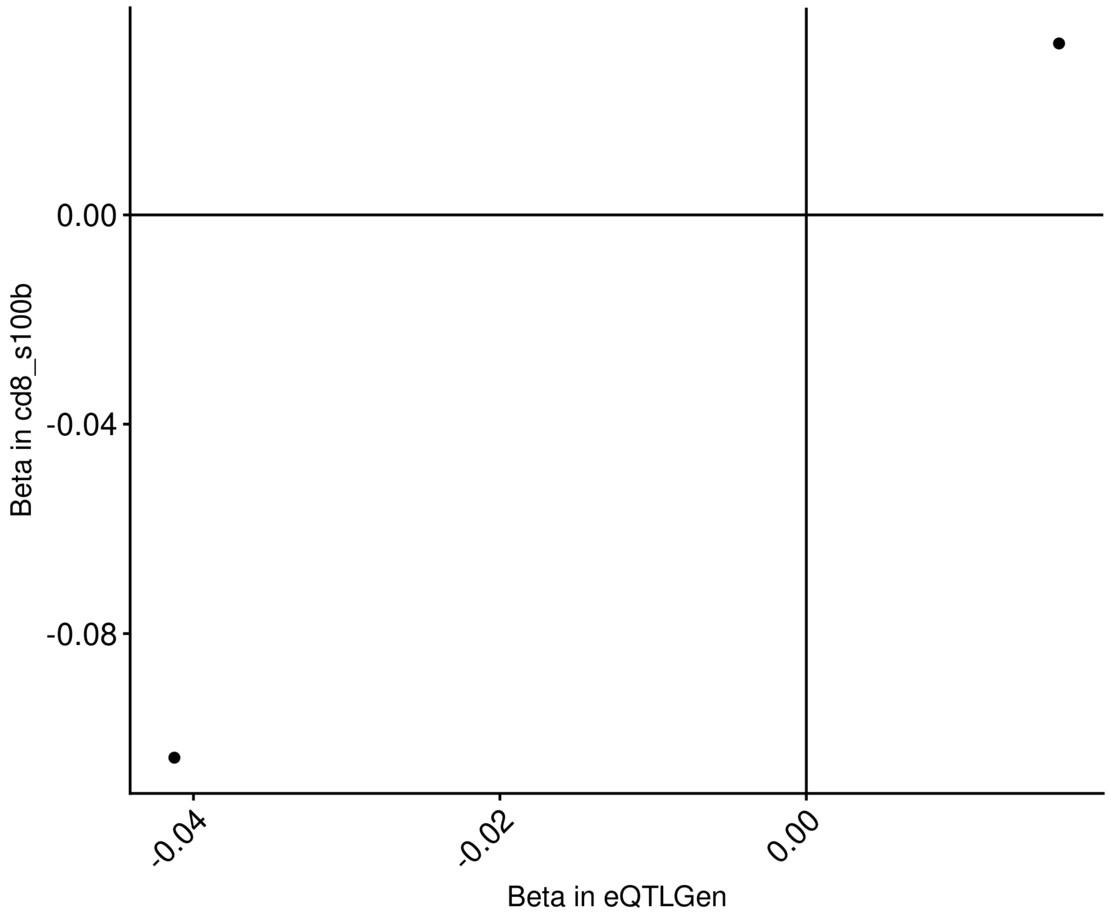

0.00
-0.04
-0.08
Beta in cd8_s100b
-0.04
-0.02
0.00
Beta in eQTLGen

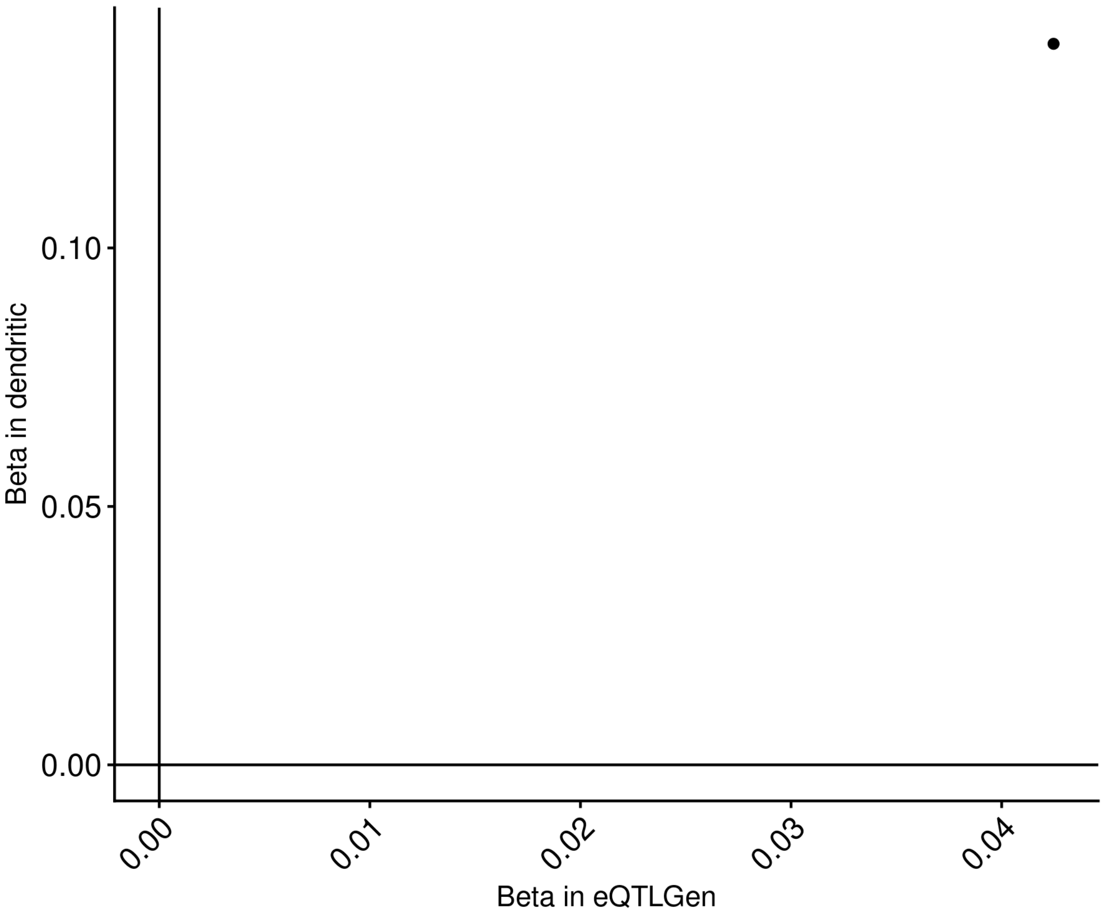
0.10
0.05
0.00
Beta in dendritic
0.00
0.01
0.02
0.03
0.04
Beta in eQTLGen

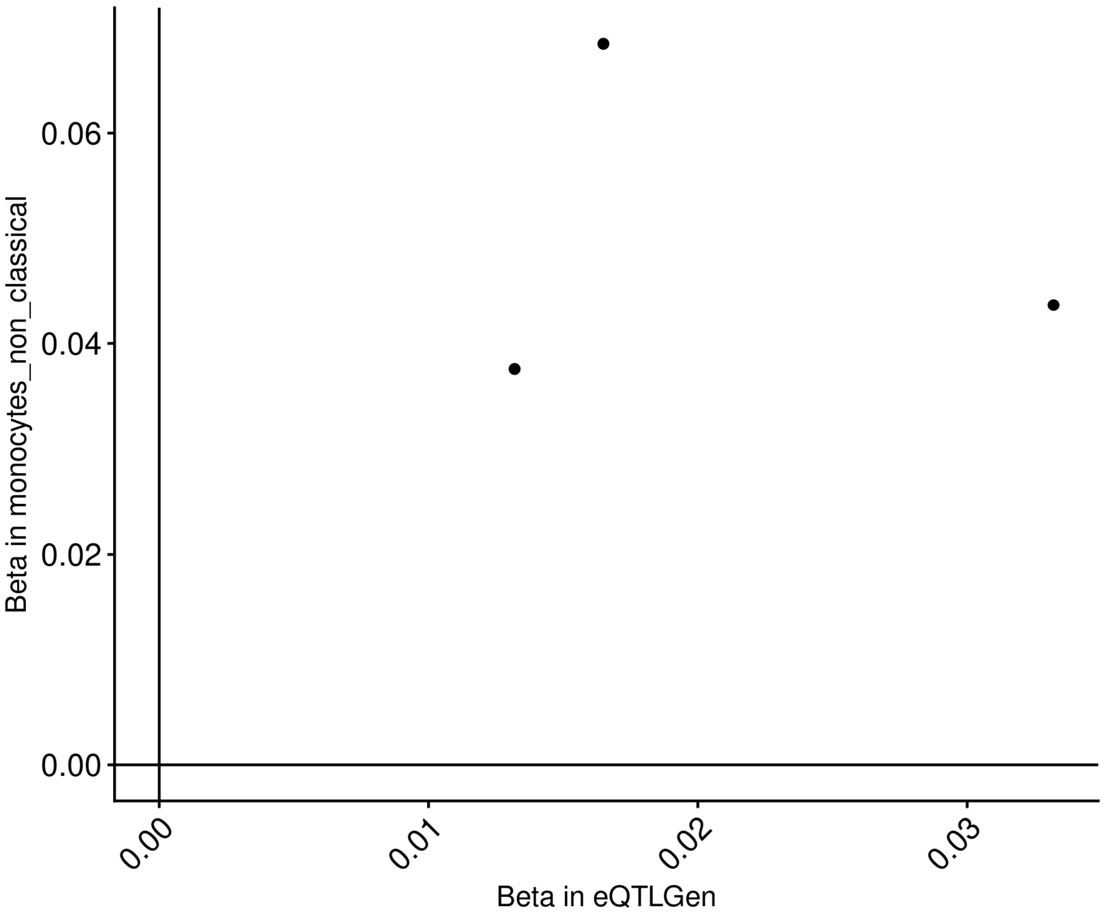
0.06
0.04
0.02
0.00
Beta in monocytes_non_classical
0.00
0.01
0.02
0.03
Beta in eQTLGen

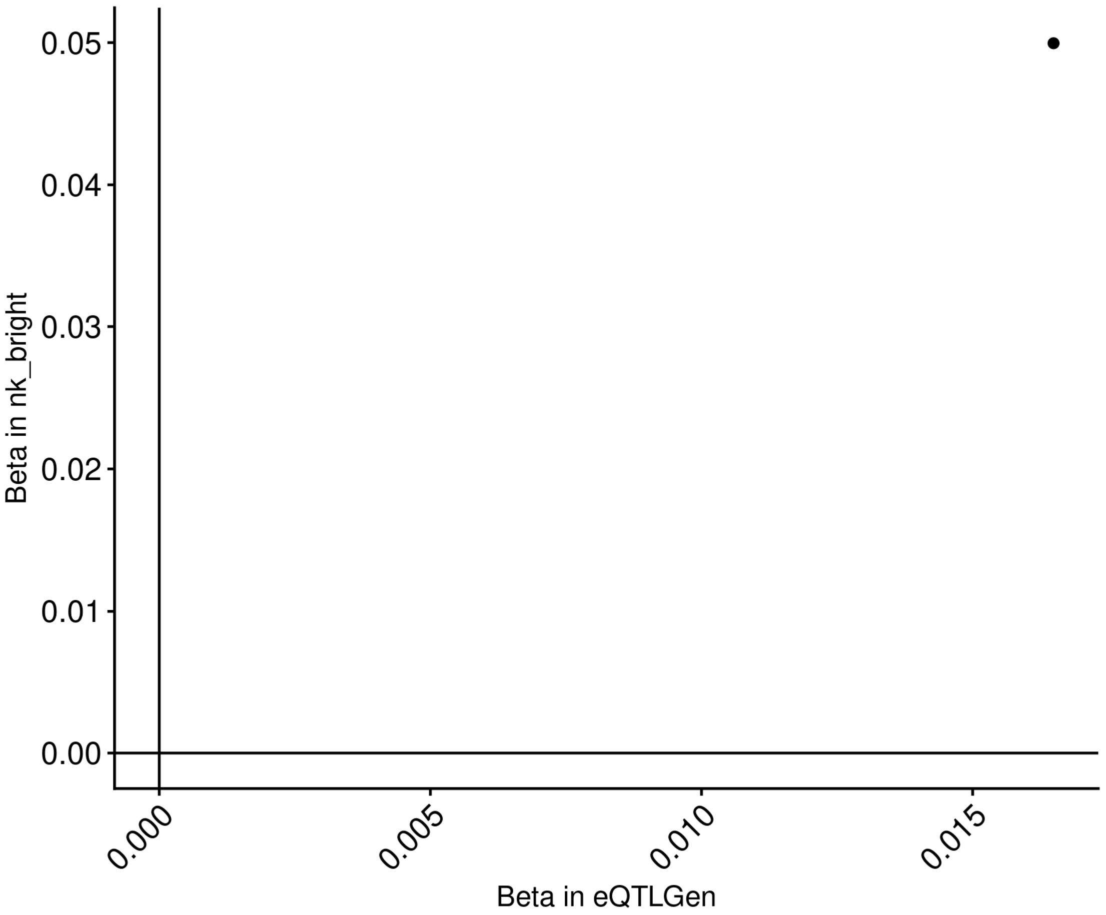

0.05
0.04
0.03
0.02
0.01
0.00
Beta in nk_bright
0.000
0.005
0.010
0.015
Beta in eQTLGen

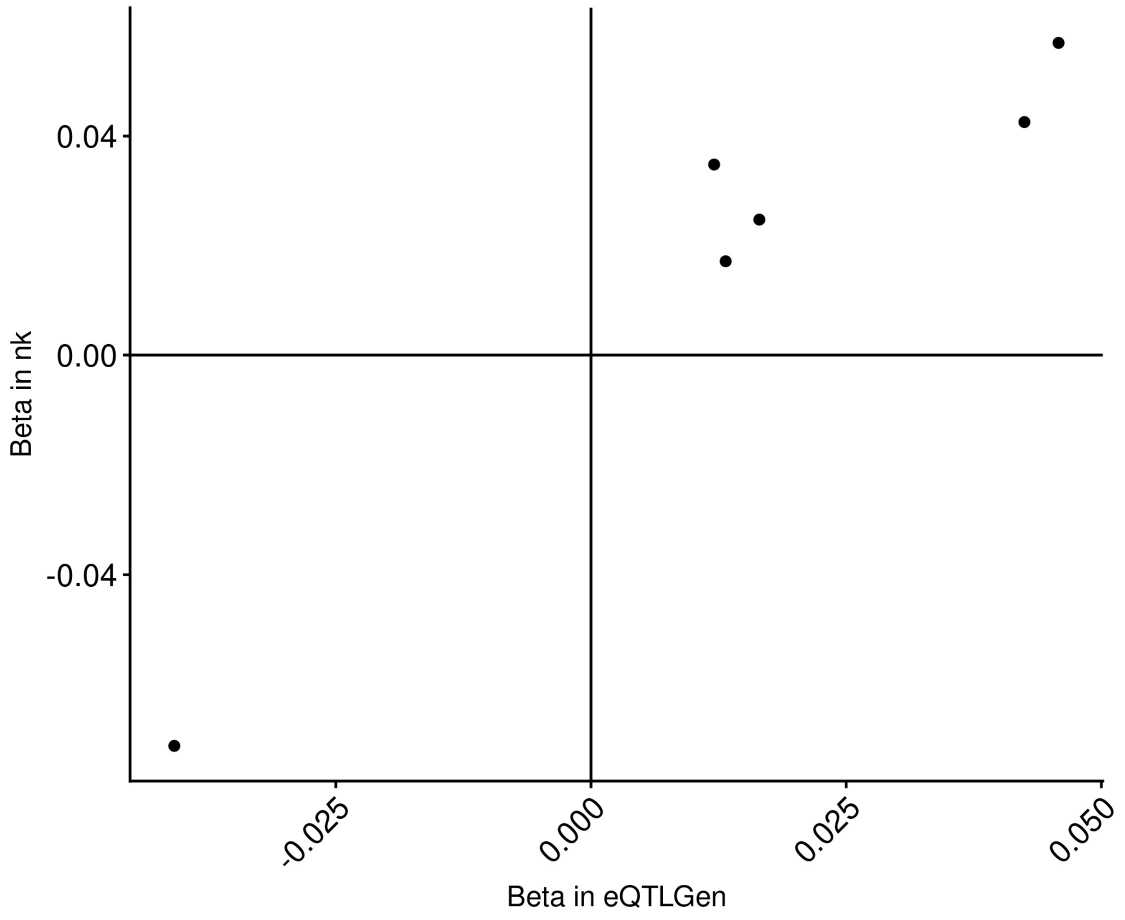
0.04
0.00
-0.04
Beta in nk
-0.025
0.000
0.025
0.050
Beta in eQTLGen

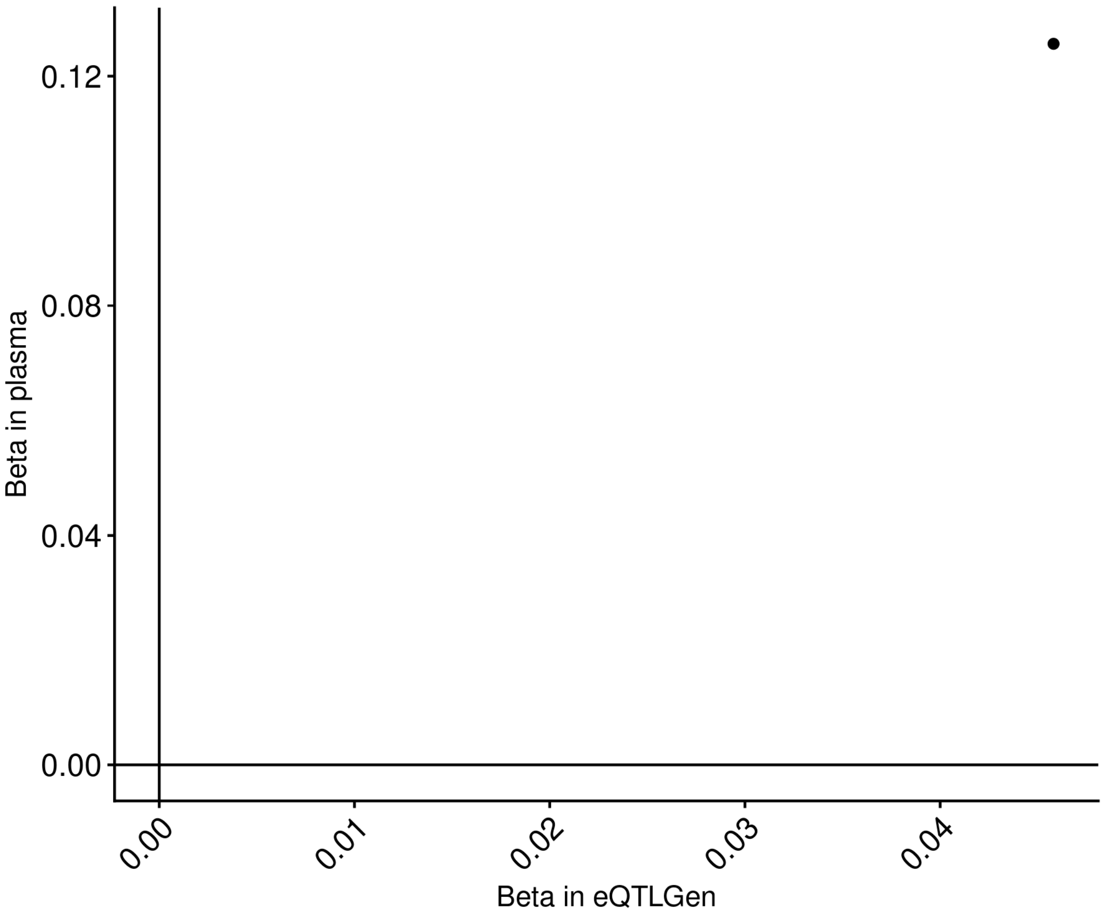
0.12
0.08
0.04
0.00
Beta in plasma
0.00
0.01
0.02
0.03
0.04
Beta in eQTLGen

Figure S5. Cell-specific effect size estimates of 22 high-confidence risk genes for AD.

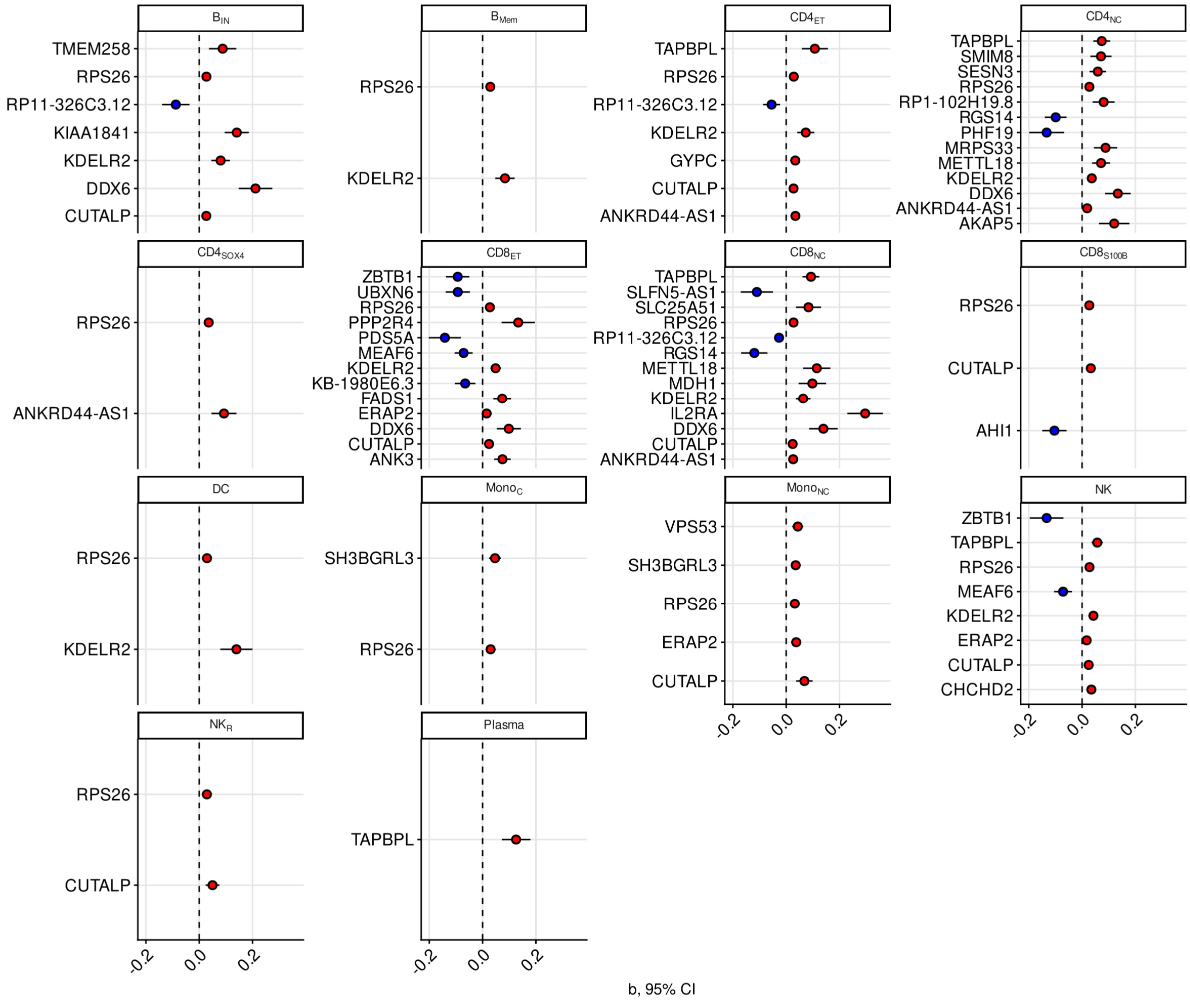

Figure S6. Pathway enrichment analysis using the g:Profiler web-based tool.

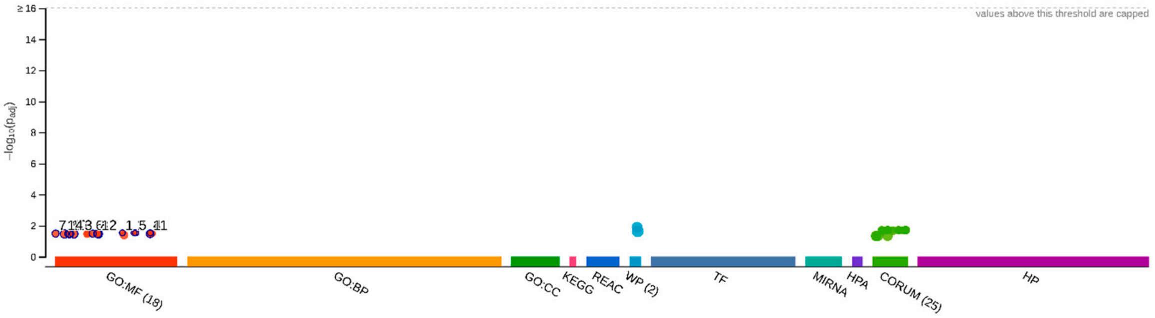

ID	Source	Term ID	Term Name	p_{adj} (query_1)
1	GO:MF	GO:0045485	omega-6 fatty acid desaturase activity	2.928×10^{-2}
2	GO:MF	GO:0047860	diiodophenylpyruvate reductase activity	2.928×10^{-2}
3	GO:MF	GO:0062076	acyl-CoA (8-3)-desaturase activity	2.928×10^{-2}
4	GO:MF	GO:0062062	oligosaccharyltransferase complex binding	2.928×10^{-2}
5	GO:MF	GO:0047995	hydroxyphenylpyruvate reductase activity	2.928×10^{-2}
6	GO:MF	GO:0018064	protein-L-histidine N-tele-methyltransferase acti...	3.252×10^{-2}
7	GO:MF	GO:0000248	C-5 sterol desaturase activity	3.252×10^{-2}
8	GO:MF	GO:0019976	interleukin-2 binding	3.375×10^{-2}
9	GO:MF	GO:0005046	KDEL sequence binding	3.375×10^{-2}
10	GO:MF	GO:0004911	interleukin-2 receptor activity	3.375×10^{-2}
11	GO:MF	GO:0062061	TAP complex binding	3.375×10^{-2}
12	GO:MF	GO:0030060	L-malate dehydrogenase (NAD+) activity	3.440×10^{-2}
13	GO:MF	GO:0008160	protein tyrosine phosphatase activator activity	3.440×10^{-2}
14	GO:MF	GO:0004470	malic enzyme activity	3.440×10^{-2}

version e113_eg59_p19_6be52918
date 1/22/2026, 6:22:48 PM
organism hsapiens

g:Profiler

Figure S7. Protein-protein interaction network between high-confidence genes using both single-cell *cis*-eQTLs and bulk *cis*-eQTLs.

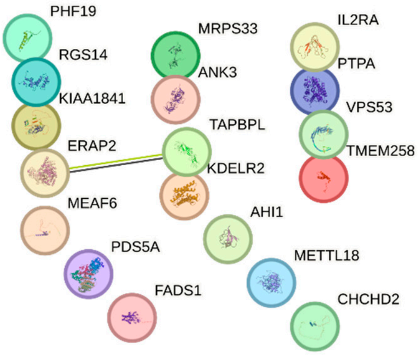

Figure S7. Protein-protein interaction network between high-confidence genes using both single-cell *cis*-eQTLs and bulk *cis*-eQTLs.

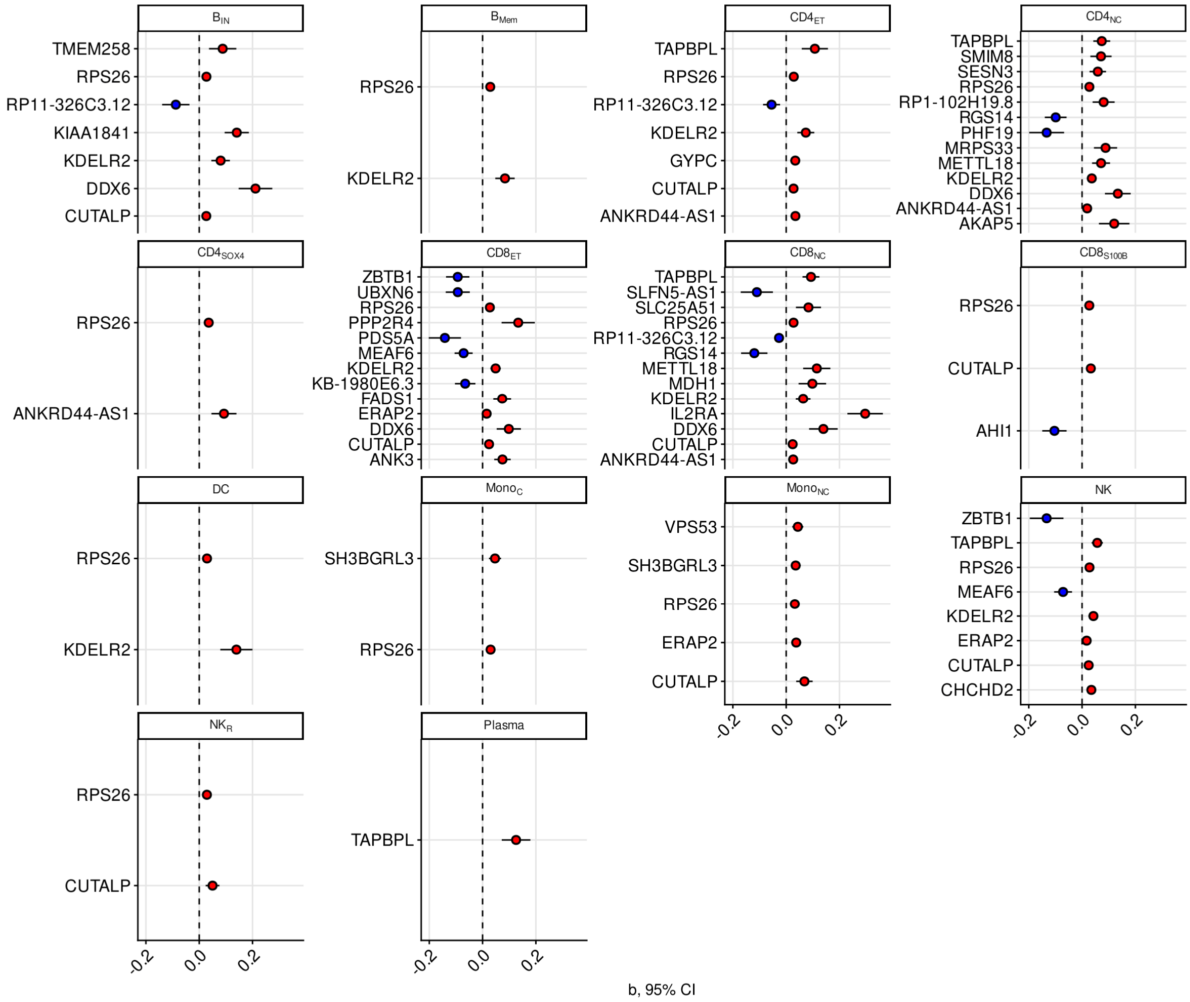

Supplement: Supplementary file 1 [file ijms-27-02226-s001.zip › Supplementary Figures.pdf]
